# Applicability of the ACE-III and RBANS Cognitive Tests for the Detection of Alcohol-Related Brain Damage

**DOI:** 10.3389/fpsyg.2019.02636

**Published:** 2019-11-28

**Authors:** Pamela Brown, Robert M. Heirene, Gareth-Roderique-Davies, Bev John, Jonathan J. Evans

**Affiliations:** ^1^Institute of Health & Wellbeing, University of Glasgow, Glasgow, United Kingdom; ^2^Graham Anderson House, Brain Injury Rehabilitation Trust, Glasgow, United Kingdom; ^3^Addictions Research Group, School of Psychology and Therapeutic Studies, University of South Wales, Pontypridd, United Kingdom; ^4^Brain and Mind Centre, School of Psychology, The University of Sydney, Sydney, NSW, Australia

**Keywords:** ARBD, Korsakoff’s syndrome, ACE-III, RBANS, diagnosis

## Abstract

**Background and Aims:**

Recent investigations have highlighted the value of neuropsychological testing for the assessment and screening of Alcohol-Related Brain Damage (ARBD). The aim of the present study was to evaluate the suitability of the Addenbrooke’s Cognitive Examination (ACE-III) and the Repeatable Battery for the Assessment of Neuropsychological Status (RBANS) for this purpose.

**Methods:**

Comparing 28 participants with ARBD (11 with Korsakoff’s Syndrome and 17 with the umbrella “ARBD” diagnosis) and 30 alcohol-dependent participants without ARBD (ALs) we calculated Area Under the Curve (AUC) statistics, sensitivity and specificity values, base-rate adjusted predictive values, and likelihood ratios for both tests.

**Results:**

High levels of screening accuracy were found for the total scores of both the ACE-III (*AUC* = 0.823, 95% CIs [0.714, 0.932], *SE* = 0.056; optimal cut-off ≤86: sensitivity = 82%, specificity = 73%) and RBANS (*AUC* = 0.846, 95% CIs [0.746, 0.947], *SE* = 0.052; optimal cut-off ≤83: sensitivity = 89%, specificity = 67%) at multiple cut-off points. Removing participants with a history of polysubstance from the samples (10 ALs and 1 ARBD) improved the diagnostic capabilities of the RBANS substantially (*AUC* = 0.915, 95% CIs [0.831, 0.999], *SE* = 0.043; optimal cut-off ≤85: sensitivity = 98%, specificity = 80%), while only minor improvements to the ACE-III’s accuracy were observed (*AUC* = 0.854, 95% CIs [0.744, 0.963], *SE* = 0.056; optimal cut-off ≤88: sensitivity = 85%, specificity = 75%).

**Conclusion:**

Overall, both the ACE-III and RBANS are suitable tools for ARBD screening within an alcohol-dependent population, though the RBANS is the superior of the two. Clinicians using these tools for ARBD screening should be cautious of false-positive outcomes and should therefore combine them with other assessment methods (e.g., neuroimaging, clinical observations) and more detailed neuropsychological testing before reaching diagnostic decisions.

## Introduction

It has been estimated that 50–80% of people who misuse alcohol will experience some degree of cognitive impairment (Bernardin et al., 2014). Deficits are primarily observed in memory, executive abilities, visuospatial processing, speed of processing and, to a lesser extent, attention and general intelligence (Stavro et al., 2013). In chronic and severe cases of alcohol-dependence, the neurocognitive impairment may progress to an extent where more debilitating and permanent damage occurs. In such cases, the person may receive a diagnosis of Alcohol-Related Brain Damage (ARBD; Royal College of Psychiatrists, 2014), or one of the more discretely defined diagnoses subsumed within this larger conceptual category such as Korsakoff’s Syndrome (see Heirene et al., 2018 for an overview of ARBD diagnoses).

Prompt recognition of ARBD is crucial to avoid further deterioration and minimize the potentially deleterious effects of cognitive dysfunction on treatment outcomes (Bates et al., 2006). Recent investigations have found that neuropsychological testing is highly effective at identifying individuals with ARBD and distinguishing them from both healthy controls and alcohol-dependent individuals with mild cognitive impairment (ALC). For example, Wester et al. (2014) found that both the Rivermead Behavioral Memory Test (RBMT; Wilson et al., 1989) and California Verbal Learning Test (CVLT; Delis et al., 1987) were useful in differentiating Korsakoff’s Syndrome (KS)—a chronic form of ARBD characterized by severe episodic memory deficits—from an ALC group, with statistically significant group differences on every index of both tests. Wester et al. (2013a) also found the updated RBMT-3 demonstrated high sensitivity and specificity values when distinguishing between KS and ALC groups, and between the latter group and healthy controls.

Brief cognitive screening tests have also proved useful for this purpose. Wester et al. (2013b) found the Montreal Cognitive Assessment (MoCA; Nasreddine et al., 2005) was able to significantly differentiate between KS and ALC groups and between KS and healthy controls, both with high sensitivity and specificity values. What is more, when all of these participants were ranked according to their RBMT-3 memory score, the MoCA was also able to differentiate between those classified as severely impaired and those deemed unimpaired, between severely and mildly impaired groups, and between mild and unimpaired groups all with good sensitivity and specificity. Oudman et al. (2014) have also compared the ARBD screening properties of the MoCA with the Mini-Mental Status Examination (MMSE; Folstein et al., 1975). Comparing KS and controls, both screening tests were able to significantly differentiate between the groups with high sensitivity and specificity, though the MoCA was the superior of the two.

Several other cognitive tests have been used to assess ARBD but are yet to be specifically evaluated for this purpose. In particular, the Addenbrooke’s Cognitive Examination-III (ACE-III; Hsieh et al., 2013) and Repeatable Battery for the Assessment of Neuropsychological Status (RBANS) have been used repeatedly to assess cognitive impairments in alcohol-dependent individuals both with (e.g., Spiegel and Jim, 2011; Wilson et al., 2012) and without (e.g., Green et al., 2010; Rao, 2016) ARBD. Both the ACE-III and RBANS, whilst comparatively short compared with batteries of global function such as the Wechsler Adult Intelligence Scale, provide a more extensive assessment of cognition than screening tests such as the MMSE or MoCA. The combination of being relatively brief but providing a more thorough assessment of cognitive function may make these tools particularly suitable for use in alcohol treatment services or hospital wards where time restrictions often apply, yet a more detailed assessment is warranted than that provided by a screening tool such as the MMSE or MoCA.

The ability of the previous version of the ACE-III, the ACE-R, to identify cognitive impairments in alcohol-dependent individuals without ARBD diagnoses was recently found to be comparable to the MoCA and superior to the MMSE (Ridley et al., 2017). The RBANS too, appears useful for identifying alcohol-related cognitive impairments. Green et al. (2010) found large effect sizes for comparisons between alcohol-dependent individuals and controls on tests of immediate memory, visuospatial abilities, and the overall test score. However, the authors highlighted the RBANS’s inadequate assessment of executive abilities as a limitation of the test. The same criticism could also be said of the ACE-III, which assess only one function classified under the rubric of executive function: verbal fluency. This may restrict the use of both tests with this population, as varying degrees of executive dysfunction have become recognized as a central feature of ARBD (van Oort and Kessels, 2009; Maharasingam et al., 2013). Nonetheless, the accuracy of these tests for ARBD screening remains unknown. Indeed, in a recent systematic review of studies evaluating the value of multiple neuropsychological tests in the assessment of alcohol-related cognitive impairment (Heirene et al., 2018), the authors highlighted the ACE-III and RBANS as two tests requiring further validation for ARBD assessment. Moreover, in a recently completed prevalence study conducted by some of the present authors (under review), the ACE-III and RBANS were the most commonly used cognitive tests in the diagnosis of ARBD in the United Kingdom. As a result, the aim of this study was to evaluate the psychometric and diagnostic validity of the ACE-III and RBANS for ARBD screening, and to compare the relative value of the two tests for this purpose.

In the present study, a group of persons with ARBD diagnoses was compared to alcohol-dependent individuals with no such diagnosis (ALs). This comparison group was selected instead of healthy controls as clinicians involved in the assessment of cognition in alcohol-dependent individuals are likely to be more focused on establishing whether the impairment is clinically significant (i.e., likely to have a substantial impact on the person’s ability to function on a day-to-day basis), as opposed to its absence or presence. The ability to differentiate between these two groups has important clinical implications as those with ARBD may require the addition of cognitive rehabilitation strategies to their treatment (Svanberg and Evans, 2013), as well as the implementation of strategies to compensate for cognitive deficits (Arts et al., 2017). Thus, if the two screening tests can differentiate those with ARBD from those without, as has been found for other commonly used screening measures (i.e., MoCA and MMSE), then they can provide quick and relatively inexpensive methods of identifying those who may require more support than offered by traditional treatments.

## Materials and Methods

### Participants

A total of 60 persons agreed to participate in the study (AL: *n* = 31, ARBD: *n* = 29). Prior to data collection, a power analysis was performed using G^∗^Power (Faul et al., 2007) to determine adequate sample size. Studies using the ACE-III and RBANS to assess alcohol-related cognitive deficits are scant, though Green et al. (2010) found large effect sizes (*d* = 1.08—1.17) on 3 of the RBANS’ scores between controls and moderate-heavy alcohol consumers. Similarly, comparisons of the ACE-III total score between cognitively impaired substance misusers (mostly alcohol-related) and unimpaired controls in Ridley et al. (2017) produced a very large effect size (*d* = 1.42; calculated by the present authors based on descriptive statistics provided by Ridley et al., 2017). Based on this demonstrated sensitivity to alcohol-related cognitive deficits, a power calculation for a one-tailed independent pairs t-test with an estimated medium-large effect size (0.7; Cohen’s *d*), alpha at 0.5, and power at 0.8 estimated that 26 participants would be required in each group. A satisfactory sample size was therefore achieved for both groups.

All ARBD participants were recruited through the Glasgow specialist ARBD service. AL participants were recruited from community rehabilitation services (i.e., non-profit addiction support agencies; *n* = 20), hospital day-patient services (e.g., psychiatry, occupational therapy; *n* = 5), and secondary services (e.g., community addictions services, psychology; *n* = 5). The ARBD group comprised 11 persons with a diagnosis of KS and 18 with the umbrella diagnostic term “ARBD.” This latter diagnostic conceptualization has been exposited by Wilson (2013) and Wilson et al. (2011), who propound an inclusive and pragmatic approach to the nosology of alcohol-related neurocognitive decline which has now been adopted in United Kingdom clinical practice. According to Wilson and colleagues, a person with ARBD must meet two key criteria: [1] evidence of cognitive impairment (as demonstrated by clinical examination or cognitive testing) and [2] a significant history of alcohol misuse (i.e., a minimum average of 35 standard drinks per week for men and 28 for woman for a period of 5 years). The authors have also proposed several other symptoms and behaviors that may support the presence of ARBD (e.g., neuroimaging evidence of cerebellar atrophy; frequent and/or delayed hospital admissions attributable to their alcohol use or social and/or psychiatric problems), as well as those that may indicate the presence of complicating conditions (e.g., neuroimaging evidence of cortical or subcortical infarction, subdural hematoma or other focal brain pathology).

The origin of participants’ diagnoses varied and therefore the exact procedures used to make diagnostic decisions was unknown. However, in the Glasgow area, clinicians report that ARBD diagnoses are—in line with Wilson and colleagues’ criteria (2011; 13)—typically made according to most or all of the following criteria: [1] chronic and excessive alcohol history, [2] evidence of cognitive deficits typically associated with alcohol-dependence (e.g., impairments in episodic and working memory, verbal fluency, and visuospatial processing), [3] neuroimaging evidence of structural brain change, and [4] psychosocial deterioration. KS diagnoses are typically made based on the ICD-10 criteria for Alcohol-Related Amnesic Syndrome (World Health Organisation, 1992), and also involves a combination of assessing alcohol-use history, neuropsychological testing, neuroimaging, and general clinical examination. All AL participants met ICD-10 criteria for Dependence Syndrome and had no evidence of ARBD. All diagnoses were made independently of results from either the ACE-III or RBANS and made by clinicians who were not part of the research team.

For inclusion in the study, participants were required to be abstinent from alcohol and other substances (excluding caffeine and nicotine) for a minimum of five-weeks at testing and have no serious physical (e.g., severe hepatic disease) or psychological (e.g., schizophrenia) complications. Less severe psychological disorders (e.g., mild anxiety or depression) were not criteria for exclusion. Due to the high incidence of head injuries in this population it was considered unrealistic to exclude individuals who had experienced *any* head injury. However, evidence of severe brain injury (Glasgow Coma Scale: 3–8 (Teasdale and Jennett, 1974); Post-Traumatic Amnesia >7 days; loss of consciousness: >24 h) or previous cranial surgery were used as exclusion criteria. To meet the requirements of testing, all participants were required to have use of their dominant hand, adequate visual function, and not suffer from receptive or expressive aphasia. Based on these criteria, one AL participant was excluded for not meeting the minimum abstinence requirement and one individual with an ARBD diagnosis was excluded due to neuroimaging evidence of intracranial hemorrhage within both frontal lobes resulting from a traumatic brain injury.

The demographic and clinical characteristics of the final 58 participants are displayed in Table 1. Groups were approximately matched for gender, medication use, and occupational status distribution. The ARBD group were significantly older, had a significantly longer duration of abstinence, were more likely to have suffered from previous head injuries, and had longer drinking histories on average; although these latter two differences were not statistically significant. The AL group contained significantly more individuals with a history of polysubstance use (defined as using any illicit substance other than cannabis [which was common among both groups] on more than one occasion). Varying types and degrees of other-substance use were reported by polysubstance users, including the use of heroin, amphetamines, crack cocaine, cocaine, ecstasy, and diazepam; still, alcohol was the primary substance used by all.

**TABLE 1:** Characteristics of AL and ARBD participants.

	**Group**		** Comparison**
		
	**AL**	**ARBD**	
*n*	30	28 (KS = 11; ARBD = 17)	
Gender: male/female	19/11	18/10	χ^2^ = 0.057, *p* = 0.940
Age in years: *M* (*SD*)	46.1 (8.9)	56.9 (7.2)	*t* = 5.084, *p* < 0.001
Weeks of abstinence: *Mdn* (*range*)	19.5 (5–722)	70.5 (9–416)	*U* = 207, *p* < 0.001
Drinking history (years): *Mdn* (*range*)	16 (3–55)	20 (2–40)	*U* = 354, *p* = *0.303*
Poly-substance use history: *n* (%)	10 (33.3)	1 (3.5)	χ^2^ = 8.35, *p* = *0.004*
Known previous head injury: *n* (%)	1 (0.33)	6 (17.9)	χ^2^ = 3.29, *p* = *0.070*
**Current medication use: *n* (%)**			
Antidepressants	17 (56.7)	15 (53.6)	χ^2^ = 0.056, *p* = 0.813
Benzodiazepines	2 (6.7)	1 (3.6)	χ^2^ = 0.283, *p* = *0.595*
Antipsychotics	2 (6.7)	3 (10.7)	χ^2^ = 0.301, *p* = 0.583
Disulfiram, naltrexone, acamprosate	6 (20.0)	2 (7.1%)	χ^2^ = 2.01, *p* = *0.156*
**Occupational status^a^: *n* (%)**			
Higher	4 (13.3)	4 (14.3)	χ^2^ = 0.251, *p* = 0.882
Intermediate	6 (20.0)	7 (25.0)	
Lower/unemployed	20 (66.7)	17 (60.7)	

**t* = Welch’s *t*-test; *U* = Mann-Whitney *U* test (employed when data did not meet parametric assumption of normal distribution); all *t*-tests and Man-Whitney *U* tests were two-tailed; χ^2^ = Chi-square test (Fisher’s exact *P* value presented where expected frequencies were < 5). ^*a*^The National Statistics Socio-Economic Classification (Rose et al., 2005) was used to classify participants according to their (previous or current) occupational status.*

### Measures

The ACE-III tests five cognitive domains: attention, memory, verbal fluency, language, and visuospatial function. Individual sub-test scores can be calculated as well as a total composite score which has a maximum of 100. Two optimal cut-off points for dementia have been identified that produce high levels of sensitivity and specificity, 88 and 82, with the former resulting in superior sensitivity to specificity and the latter the obverse (Hsieh et al., 2013; So et al., 2018). Administration and scoring takes between 15 and 20 min and does not require specialist training in psychometric testing; although introductory training and familiarization with the test before use is recommended. The test is currently available for free in 30 languages, in iPad and mobile form, and in a miniature version. The Brain and Mind Centre at the University of Sydney provide all versions of the test for free, along with administration guidance^1^.

The RBANS contains 12 subtests which provide five index scores: Immediate memory, Visuospatial/constructional, Language, Attention, and Delayed memory. Combining these index scores provides an overall performance score. All scores are converted to age-adjusted norm scores which have a mean of 100 and SD of 15. Normative data is available for participants aged 12–89 years and four parallel versions of the test have been developed for repeat testing, with forms A and B adapted to United Kingdom use. Administration of the test typically takes 20–30 min. The test is available in over 20 languages and can be purchased from Pearson Clinical Assessments. Pearson state that the test can be used by allied health or special educational professionals, as well as those with more formal training in psychometric assessment.

### Procedures

Potential participants were provided with written details of the study by a professional contact (e.g., support worker, care manager) and asked to arrange an appointment. All individuals meeting inclusion criteria were fully informed of the study’s procedures and provided written consent to participate and for access to medical records to ensure there were no significant complications which would preclude participation. Individuals with ARBD were assessed within residential units, acute settings, or within their own home. AL participants were all assessed in day-patient settings (e.g., community addictions unit). The order in which the two tests were presented was counterbalanced to avoid order effects. Ethical approval was obtained from Greater Glasgow and Clyde Health Board before commencing the study (IRAS project ID: 155916).

### Data Analysis

Analyses were conducted using jamovi (The jamovi project, 2019) and NCSS 2019. To enhance the reproducibility and transparency of the analysis, the code used in jamovi to analyze the data can be accessed via this project’s Open Science Framework (OSF) page^2^ and in Supplementary Document 1. This document also includes the full outcomes of all analyses, including tests of statistical assumptions. Sensitivity and specificity analyses were conducted using NCSS (2019) and the associated AUC graphs were made using GraphPad (version 8), therefore no code is available for these.

A combination of parametric and non-parametric tests was used for between-group comparisons, the latter whenever data were not normally distributed (Shapiro-Wilk’s test). For comparisons using parametric tests, Welch’s *t*-test was used as opposed to Student’s *t* as it is more robust to violations of homogeneity of variance and more suitable when sample sizes are uneven (see Delacre et al., 2017). As we made multiple comparisons between the AL and ARBD groups on test scores, we attempted to reduce the family-wise error rate by using a Bonferroni correction. Dividing 0.05 by the number of score comparisons (*n* = 12) resulted in an adjusted alpha of 0.0042 for these comparisons.

Receiver Operator Characteristic (ROC) analyses were conducted to determine the relative screening accuracy of the ACE-III and RBANS. The ROC analysis provides sensitivity (proportion of those with the disorder correctly identified as impaired on the test) and specificity (proportion of those without the disorder correctly identified as unimpaired on the test) values, as well as an Area Under the Curve (AUC) statistic. The AUC statistic varies between 0.5 and 1, with 1 representing perfect sensitivity and specificity. Positive and Negative Predictive Values (PPV/NPV) were also calculated to further evaluate the clinical utility of the tests. The PPV is the percentage of persons with a “positive” test score (i.e., within the impaired range) who actually have the disorder (ARBD), and NPV is the percentage with a “negative” score (i.e., within the normal range) who do not have the disorder. Oudman et al. (2014) calculated the PPV and NPV for the MMSE and MoCA, finding excellent predictive values for both tools. However, their calculations did not reflect the base-rate (prevalence) of ARBD in clinical settings, which directly influences predictive values. In order to account for this, PPVs and NPVs were calculated for the tests according to estimations of ARBD prevalence (base-rate) within the alcohol-dependent population. The proportion of alcohol-dependent individuals believed to experience some form of major neurocognitive disorder ranges from 12.5% (Zahr et al., 2011) up to 35% (Cook et al., 1998). Accordingly, predictive values were calculated for base-rates of 12.5% and 35% to reflect environments where ARBD diagnoses are likely to be queried. Finally, positive and negative likelihood ratios were calculated, which express the probability of having the condition given a positive test score and not having the condition given a negative test score, respectively.

In accord with Simmons et al. (2012), we have reported how we determined our sample size, all data exclusions, all manipulations, and all measures used in the study.

## Results

Between-group comparisons for all test scores are presented in Table 2, along with standardized (Cohen’s *d*, pooled *SD* used as the standardizer) and unstandardized effect sizes to provide a detailed understanding of findings (Lakens, 2013; Pek and Flora, 2018). The ARBD group scored significantly lower than ALs on all test indices apart from the Attention and Visuospatial scores of both tests; although, these differences (excluding that related to the ACE-III visuospatial score) approached our adjusted alpha level of 0.0042. According to Cohen’s (1992) classification of effect sizes (i.e., small: *d* = 0.2, medium: *d* = 0.5, large: *d* ≥ 0.8), large effects were observed on the ACE-III for the Total score, Attention, Memory and Fluency, and small effects for Language and Visuospatial scores. For the RBANS, large effects were observed for Total score, Immediate Memory, and Delayed Memory, while medium effects were found for Visuospatial, Language and Attention.

**TABLE 2:** ACE-III and RBANS performance by alcohol-dependent individuals with and without ARBD.

	**Index (maximum score)**	***M* (*SD*), *Mdn***		**Comparison**	***M* diff [95% CIs]^a^**	**Cohen’s *d***
					
		**AL**	**ARBD**			
ACE-III	Total score (100)	89.4 (8.90), 91.0	78.5 (10.3), 80.0	*U* = 149, *p* < 0.001	10.9 [5.80, 15.9]	1.14
	Attention (18)	17.1 (1.17), 18.0	15.0 (2.97), 16.0	*U* = 225, *p* < 0.001	2.10 [0.88, 3.32]	0.94
	Memory (26)	21.3 (3.98), 22.0	16.2 (4.57), 16.5	*U* = 156, *p* < 0.001	5.12 [2.86, 7.39]	1.20
	Fluency (14)	12.0 (2.26), 13.0	9.79 (2.56), 10.0	*U* = 206, *p* < 0.001	2.21 [0.94, 3.49]	0.92
	Language (26)	24.3 (2.85), 25.0	23.5 (1.60), 24.0	*U* = 239, *p* = 0.002	0.80 [−0.41, 2.01]	0.34
	Visuospatial (16)	14.7 (1.29), 15.0	14.1 (1.84), 14.0	*U* = 341, *p* = 0.104	0.63 [−0.22, 1.48]	0.40
RBANS	Total score	89.8 (16.1), 91.5	69.2 (11.6), 68.5	*t* = 5.62, *p* < 0.001	20.6 [13.3, 28.0]	1.46
	Immediate memory	89.1 (18.7), 87.0	64.9 (13.7), 65.0	*t* = 5.65, *p* < 0.001	24.2 [15.6, 32.8]	1.47
	Visuospatial	96.5 (20.8), 92.0	83.8 (17.8), 84.9	*t* = 2.52, *p* = 0.007	12.8 [2.62, 22.9]	0.66
	Language	93.4 (11.5), 94.5	87.0 (7.10), 86.0	*U* = 229, *p* = 0.002	6.40 [1.41, 11.4]	0.67
	Attention	91.0 (15.5), 89.5	80.4 (16.0), 82.0	*t* = 2.55, *p* = 0.007	10.5 [2.25, 18.8]	0.67
	Delayed memory	91.8 (15.9), 94.0	62.8 (18.2), 58.0	*t* = 6.44, *p* < 0.001	29.0 [20.0, 38.0]	1.70

*All tests one-tailed (ARBD < AL); Mean differences (*M* diff) and 95% CIs for Mann Whitney-*U* analyses are presented from Welch’s *t* outcomes for consistency with parametric test outcomes. Means and medians (*Mdn*) are provided for consistency across outcomes and to present the most complete picture of findings. ^*a*^Two-tailed confidence intervals presented to provide upper limits.*

As we identified multiple statistically significant differences between the groups for the variables presented in Table 1, we checked the robustness of the between-group differences on each test score by running analyses of covariance (ANCOVAs) for each comparison and including age, weeks of abstinence, and polysubstance use history (coded as a categorical variables with “yes” or “no” outcomes) as covariates in the models. All statistically significant comparisons between groups at *p* < 0.004 reported in Table 2 remained significant at this adjusted alpha level aside from the Fluency (*p* = 0.012) and Language (*p* = 0.086) scores of the ACE-III. Thus, when accounting for between-group differences in demographic and clinical variables, it appears that discrepancies in memory scores most differentiate the groups on both tests. The full outcomes for all 12 ANCOVAs and the analysis code used to produce them in jamovi are available on OSF (see text footnote 2) and in Supplementary Document 1.

Several exploratory analyses were conducted on total test scores to explore possible within-group differences. Alpha was set at 0.05 to minimize the risk of false negative outcomes (i.e., Type-II errors; see Hartgerink et al., 2017; Witt, 2019). The potential adverse consequences associated with false-positive outcomes (i.e., Type-I) in these exploratory analyses was deemed to be low (Lakens et al., 2018). Nonetheless, the following outcomes should be interpreted as reflecting exploratory, preliminary evaluations of the data. First, due to the high number of ALs with a history of polysubstance use, a within-group comparison between those with a history of polysubstance use and those without was undertaken. No significant difference was observed between AL polysubstance users (*n* = 10, *M* = 85.5, *SE* = 3.5) and ALs who only used alcohol (*n* = 20, *M* = 91.3, *SE* = 1.6) for the ACE-III total score, *t*(12.9) = 1.52, *p* = 0.152, *d* = 0.68. On the RBANS, however, a significant difference was found between those with a polysubstance use history (*M* = 78.2, *SE* = 4.1) and those without (*M* = 95.7, *SE* = 3.2) for the total score, *t*(20.12) = 3.36, *p* = 0.003, *d* = 1.25. These poorer scores could not be explained by differences in drinking history duration (*U* = 83, *p* = 0.466) or length of abstinence (*U* = 75.5, *p* = 0.289) between the two sub-groups. The ARBD group was also dichotomized for further analysis into individuals with a specific diagnosis of KS and those with the broad “ARBD” diagnosis. No significant difference was found between individuals with KS (*n* = 11; ACE-III: *M* = 77.1, *SE* = 3.5; RBANS: *M* = 65.7, *SE* = 2.7) and those with ARBD (*n* = 17; ACE-III: *M* = 79.5, *SE* = 2.3; RBANS: *M* = 71.5, *SE* = 3.1) for ACE-III, *t*(18.4) = 0.568, *p* = 0.577, or RBANS scores, *t*(25.9) = 1.4, *p* = 0.173.

**FIGURE 1:**
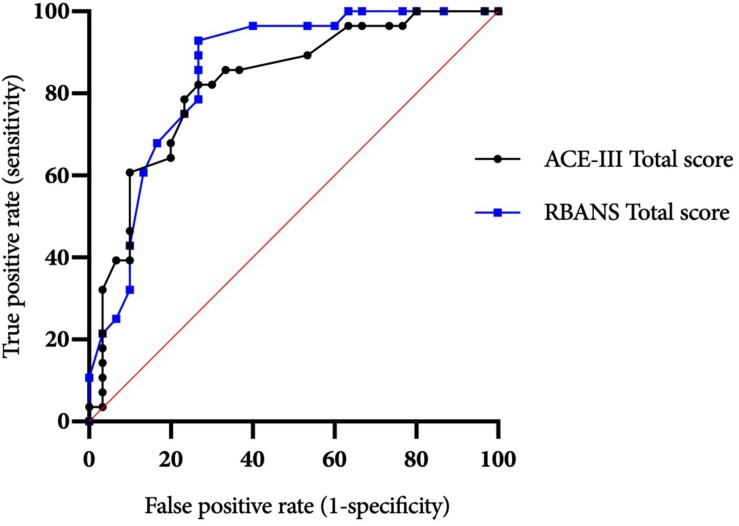
ACE-III and RBANS ROC curves for differentiating between alcohol-dependent individuals with and without ARBD.

ROC curves for the ACE-III and RBANS are displayed in Figure 1. Table 3 displays test cut-off scores and their corresponding diagnostic values, including the number of AL and ARBD participants classed as impaired using this cut-off, positive and negative predictive values, and positive and negative likelihood ratios. Only cut-off scores that produced optimal sensitivity (≥ 80%) and specificity (≥ 60%) levels are presented, consistent with previous research in this area (Oudman et al., 2014). The higher threshold for sensitivity over specificity is consistent with the view that screening tests should have high levels of sensitivity to maximize disease detection, while subsequent assessments should have high levels of specificity in order to ensure accurate diagnosis and avoid misdiagnosis (McNamara and Martin, 2018).

**TABLE 3:** Diagnostic validity of the ACE-III and RBANS for differentiating between alcohol-dependent individuals with and without ARBD.

	**Comparison**	**Cut-off**	**Sensitivity/specificity**	**No. ARBD/AL classified as impaired (%)**	**PPV (base-rate)**		**NPV (base-rate)**		**PLR**	**NLR**
								
					**(12.5%)**	**(35%)**	**(12.5%)**	**(35%)**		
ACE-III	ARBD vs. AL	**86**	**82/73**	**23 (82)/8 (27)**	**30.1**	**62.4**	**96.6**	**88.4**	**3.08**	**0.24**
		87	82/70	23 (82)/9 (30)	28.1	59.6	96.5	87.9	2.80	0.26
		88	86/67	24 (86)/10 (33)	26.9	58.1	97.0	89.7	2.60	0.21
		90	86/63	24 (86)/11 (37)	25.0	55.7	96.9	89.2	2.32	0.23
	Excluding polysubstance users: ARBD (*n* = 27) vs. AL (*n* = 20)	86	81/75	22 (81)/5 (25)	31.8	63.7	96.6	88.3	3.26	0.25
		**88**	**85/75**	**23 (85)/5 (25)**	**32.7**	**64.7**	**97.3**	**90.4**	**3.41**	**0.20**
RBANS	ARBD vs. AL	81	82/70	23 (82)/9 (30)	28.1	59.6	96.4	87.9	2.74	0.26
		82	86/67	24 (86)/10 (33)	26.9	58.1	97.0	89.7	2.60	0.21
		**83**	**89/67**	**25 (89)/10 (33)**	**27.7**	**59.1**	**97.8**	**92.0**	**2.70**	**0.16**
		84	89/63	25 (89)/11 (37)	25.8	56.7	97.6	91.7	2.44	0.17
		85	96/63	27 (96)/11 (37	27.3	58.6	99.2	97.1	2.63	0.06
		86	96/60	27 (96)/12 (40)	25.6	56.5	99.1	96.9	2.41	0.06
	Excluding polysubstance users: ARBD (*n* = 27) vs. AL (*n* = 20)	81	81/80	22 (81)/4 (20)	36.8	68.7	96.8	88.9	4.10	0.23
		82	85/80	23 (85)/4 (20)	37.8	69.6	97.4	90.9	4.26	0.19
		83	89/80	24 (89)/4 (20)	38.8	70.5	98.1	93.0	4.44	0.14
		**85**	**96/80**	**26 (96)**/4 (20)	**40.1**	**72.2**	**99.3**	**97.6**	**4.81**	**0.05**
		86	96/75	26 (96)/5 (25)	35.5	67.5	99.3	97.4	3.85	0.05
		87	96/70	26 (96)/6 (30)	31.4	63.4	99.3	97.2	3.21	0.05
		91	96/65	26 (96)/7 (35)	28.2	59.7	99.2	97.0	2.75	0.06
		95	100/60	27 (100)/8 (40)	26.3	57.4	100	100	2.50	0.00

*Emboldened cut-off scores produced the greatest sensitivity/specificity combination. Higher PPV and NPVs indicate better diagnostic accuracy (maximum = 1). Higher PLRs and lower NLRs indicate better diagnostic accuracy. PPV and NPV, positive and negative predictive values; PLR and NLR, positive and negative likelihood ratios.*

**FIGURE 2:**
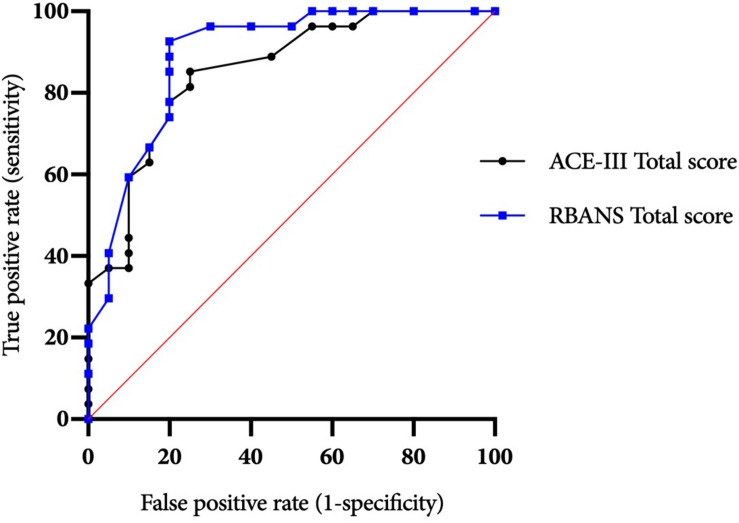
ACE-III and RBANS ROC curves for differentiating between alcohol-dependent individuals with and without ARBD (excluding polysubstance users).

The ACE-III total score was able to significantly differentiate between the AL and ARBD participants (*AUC* = 0.823, 95% CIs [0.714, 0.932], *SE* = 0.056, *p* < 0.001), with an optimal cut-off score of ≤86 producing a sensitivity of 82% and specificity of 73%. Similarly, the RBANS total score significantly distinguished between AL and ARBD participants (*AUC* = 8.46, 95% CIs [0.746, 0.947], *SE* = 0.051, *p* < 0.001), with an optimal cut-off score of ≤83 producing a sensitivity of 89% and specificity of 67%. Although the AUC value was larger for the RBANS than the ACE-III, the difference was not statistically significant (discrepancy = 0.023, *SE* = 0.045, *Z* = 0.522, *p* = 0.602). As AL participants with a history of polysubstance misuse scored significantly lower than their alcohol-use-only counterparts on the RBANS, two further exploratory ROC analyses were ran to see how sensitivity and specificity values were affected by the removal of all polysubstance users (see Figure 2 for ROC plot). This removal resulted in minor improvements to diagnostic properties of the ACE-III (*AUC* = 0.854, 95% CIs [0.744, 0.963], *SE* = 0.056, *p* < 0.001; optimal cut-off = ≤ 88: sensitivity = 85, specificity = 75) and substantial improvements to the RBANS (*AUC* = 0.915, 95% CIs [0.831, 0.999], *SE* = 0.043, *p* < 0.001; optimal cut-off: ≤85: sensitivity = 96, specificity = 80). Again, the difference between each test’s AUC value was not significant (discrepancy = 0.0611, *SE* = 0.0511, *Z* = 1.196, *p* = 0.232).

Several exploratory correlational analyses were conducted to investigate the relationships between participant characteristics (i.e., duration of drinking history, duration of abstinence, and age) and total test scores (alpha set at 0.05). None of the correlations between the groups’ test scores and drinking histories (*r*_*s*_ range = −0.023 to −0.164, *p*s ≥ 0.388), age (*r*_*s*_ range = −0.003 to −0.173, *p*s ≥ 0.378), or length of abstinence (*r*_*s*_ range = −0.253 to 0.033, *p*s ≥ 0.194) were significant [full outcomes reported on OSF (see text footnote 2) and in Supplementary Document 1]. Both groups’ total ACE-III and RBANS scores were significantly and strongly correlated (AL: *r_*s*_* = 0.784, *p* < 0.001; ARBD *r_*s*_* = 0.700 *p* < 0.001), supporting the convergent validity of the tests.

## Discussion

The present study aimed to evaluate the suitability of the ACE-III and RBANS for ARBD assessment and their ability to differentiate alcohol-dependent individuals with ARBD from those without. Both measures produced significant between-group differences on total scores and several sub- test scores, although several significant effects for the ACE-III did not remain when covariates were included in analysis models. Effect sizes (*d*) were mostly in the medium-large range, indicating a substantial discrepancy between the groups’ scores on both tests. This was particularly the case for the subtests indexing memory, which produced very large effects on both tests (*d* ≥ 1.2) that were robust to the inclusion of covariables in the models. Optimal sensitivity and specificity levels were identified for the total scores of both tools at multiple possible cut-off points. However, it should be noted that while we selected our sensitivity and specificity thresholds of ≥80 and ≥60%, respectively, for consistency with similar studies in this domain (Oudman et al., 2014), other authors have recommended optimal thresholds of ≥80% should be used for both values. Relatedly, although peak sensitivity values were high for both the ACE-III (86%) and RBANS (96%), specificity values peaked at 73% for the former and 70% for the latter, highlighting a risk of false-positives when using such cut-off scores.

Base-rate-adjusted positive and negative predictive values were also calculated for both tests at ARBD prevalence rates of 12.5 and 35%. At 12.5% prevalence, PPVs were low for both the ACE-III (peak: 30.1%) and RBANS (peak: 28.1%), further supporting a cautious interpretation of positive test scores. As would be expected, these values increased considerably when the prevalence rate was increased to 35% (ACE-III peak: 62.4%; RBANS peak: 59.6%). NPVs were high for the tests at 12.5% (ACE-III peak: 97%; RBANS peak: 99.2%) and 35% (ACE-III peak: 89.7%; RBANS peak: 97.1%) prevalence rates, supporting confident interpretations of negative test scores as true negatives. Overall, while predictive values were better overall when using the increased base-rate of 35%, this figure is predicated on the assumption that alcohol-related cerebellar degeneration is part of the same disease process as Wernicke-Korsakoff’s Syndrome (Cook et al., 1998) and may therefore be an overestimation of ARBD prevalence in the alcohol-dependent population.

Comparing the two tests, the diagnostic values produced by the ACE-III appear largely commensurate with those of the RBANS. However, some minor discrepancies between the two are evident. First, while the conflated outcomes of sensitivity and specificity values were approximately equal between the two, the ACE-III produced a higher level of specificity at its optimal cut-off (sensitivity = 82%, specificity = 73%), while the RBANS had higher sensitivity (sensitivity = 89%, specificity = 67%). This difference in ability was reflected in greater PPVs and PLRs for the ACE-III and greater NPVs and NLRs for the RBANS. Thus, the ACE-III was more likely to correctly classify those without the disorder as unimpaired than the RBANS, and the RBANS was more likely to correctly classify those with the disorder as impaired than the ACE-III. The availability of parallel versions of the RBANS may contribute to its value in assessing this population as repeat testing is required to monitor any changes cognitive dysfunction over time and in response to interventions (Royal College of Psychiatrists, 2014; Heirene et al., 2018). Thus, the RBANS can be used for the monitoring of ARBD whilst circumventing the issue of practice/learning effects associated with repeated testing. Overall, if deciding between the two, the ACE-III appears suitable when time restrictions are present, though the more extensive RBANS should be considered when time allows.

The diagnostic values of the ACE-III and RBANS found here, while high, were lower than those for the MMSE and MoCA observed by Oudman et al. (2014). However, the disparity is likely because the authors compared those with ARBD (KS) to healthy individuals – not ALs as was done here. Indeed, when Wester et al. (2013b) compared KS participants with controls the diagnostic values of the MoCA were superior to those observed here, though when KS participants were compared with an ALC group the sensitivity and specificity values did not reach optimal levels (sensitivity = 73, specificity = 75). The AL group in the present study demonstrated clear impairments relative to norm scores on both measures, suggesting similarities with the mildly impaired group studied by Wester et al. (2013b). Thus, the screening capabilities of the ACE-III and RBANS when comparing mildly versus severely impaired groups may by superior to those of the MoCA; although a direct systematic comparison would be required to confirm the superior test(s).

This is the first study to directly evaluate the screening capabilities of the ACE-III and RBANS for ARBD. Overall, the findings support the use of both tests in clinical assessments of alcohol-users; although caution should be taken to avoid false-positive tests when using the cut-off points identified. Our findings indicate that clinicians should observe individual subtests scores as well as overall scores to best differentiate those with ARBD from those without, with a particular focus on memory scores. The present study also provides a novel understanding of how using neuropsychological testing in a screening capacity for ARBD is affected by a history of polysubstance use. Findings from our exploratory analyses indicated that those with a history of polysubstance use, compared to those without such a history, will perform worse on neuropsychological tests. The poor scores by AL polysubstance users in the present study could not be explained by differences in drinking history duration or length of abstinence, suggesting it was the additional drug-use which compounded their alcohol-related cognitive deterioration; however, we cannot be certain of this from the data collected in this study. Bondi et al. (1998) reported a similar significant decrease in performance on selective tasks by ALs with concurrent polysubstance use compared to those who only used alcohol; still, causation cannot be inferred from these findings. Overall, our findings suggest a consideration of previous drug use should be made when cognitively assessing alcohol users as this may also contribute to impairment. Nonetheless, it is likely the degree of impairment, as opposed to its etiology, that is of interest to clinicians.

This is also one of the first studies to investigate the value of neuropsychological testing in the detection of ARBD, as opposed to more discretely defined diagnoses such as KS. The recent impetus for using ARBD as a broad conceptual diagnostic term has been motivated by heterogeneity within those diagnosed with KS, including varying numbers of individuals with executive (van Oort and Kessels, 2009) and/or intellectual (Jacobson and Lishman, 1987) deficits, as well as a high prevalence of head injuries, liver disease and other factors which can confound neurocognitive impairment and create further inter-person variability. Indeed, Bowden (1990) has argued that the rigid selection criteria implemented by KS researchers may render their samples artifacts of this process which are, as a result, unrepresentative of the heterogeneous presentation more typical of this population. Comparing the two diagnostic sub-divisions of the ARBD group, no statistically meaningful difference was identified between those with KS and those with ARBD on the total scores of the ACE-III or RBANS; although the sample sizes were small, potentially limiting the ability to detect any subtle differences. Due to small sample sizes, no further differences between the sub-groups’ scores were explored. Future research should compare larger samples of persons with KS and ARBD to explore whether differences in cognitive profiles underpin the choice of diagnostic nomenclature in modern clinical settings, thereby evaluating the merit of the distinction.

The primary limitation of this study was the absence of a reference standard assessment to confirm the existing diagnosis in the ARBD population. However, as previously stated, ARBD diagnoses in the study area are made against rigorous criteria by the Glasgow ARBD service which specializes in the diagnosis and treatment of those with the condition. Additionally, the ARBD group had significantly longer drinking histories and consistently poorer scores on both the ACE-III and RBANS, supporting the diagnostic distinction between groups. A second limitation was the significant difference between groups in regards to age, polysubstance use history, and weeks of abstinence. Although, to account for these differences, we ran ANCOVAs for all group comparisons and included these variables as covariates and have transparently reported all outcomes from these in addition to *t*-test and Mann-Whitney-*U* outcomes (Supplementary Document 1).

While the ACE-III and RBANS can be used to screen several different neurocognitive disorders (including mild cognitive impairment and various dementias; Karantzoulis et al., 2013; Bruno and Schurmann Vignaga, 2019), two screening tests have been developed recently specifically for assessing alcohol-related cognitive impairments. The first of these, the BEARNI (Brief Examination of Alcohol-related Neuropsychological Impairments; Ritz et al., 2015), was designed to be easily administered by non-specialists and assesses working and episodic memory, visuospatial skills, executive function, and ataxia. The second test, the TEDCA (Test of Detection of Cognitive Impairment in Alcoholism; Jurado-Barba et al., 2017), assesses working and episodic memory and visuospatial skills. Both tests may provide equally, if not superior, screening capabilities to the ACE-III and RBANS for ARBD detection, though neither test has been specifically validated for the screening of clinically diagnosed alcohol-related neurocognitive disorders (e.g., KS, ARBD). The BEARNI was found to have very high sensitivity (100%) for detecting individuals with cognitive impairment (as determine by detailed neuropsychological assessment) within a sample of ALs, although the specificity of the test was very poor (4%; Pelletier et al., 2018). In the same sample, the MoCA demonstrated 79% sensitivity and 65% specificity. Future research in this domain should focus on evaluating the screening capabilities of both tests for the populations studied here to determine whether they may better replace more generalized tests used in clinical practice (e.g., ACE-III, MoCa etc.).

In sum, the present findings add to a recent body of evidence suggesting that neuropsychological tests can be used effectively to inform the ARBD diagnostic process. The two tests studied here are more extensive in their assessment of cognition than the cognitive screening tests previously investigated for this purpose (i.e., MoCA and MMSE) and can therefore provide a more comprehensive overview of impaired and preserved cognitive abilities, whilst still remaining relatively quick to administer. Nonetheless, screening tests alone should not be used to confirm ARBD diagnoses (Heirene et al., 2018). Informed diagnostic decision making and treatment planning require more thorough assessments of cognition to detail the severity of impairment and the specific skills affected. In particular, further assessment of executive function (e.g., Behavioral Assessment of the Dysexecutive Syndrome) is warranted as both the ACE-III and RBANS lack sufficient testing of this domain. Finally, it is important to note that while neuropsychological testing is an important and informative feature of ARBD assessment, so too are clinical observations, assessments of activities of daily living, nutritional status investigations, and neuroimaging procedures (Horton et al., 2015), and thus each should be used in conjunction whenever possible.

## Data Availability Statement

Research data are not shared publicly due to stipulations made by the research ethics committee at the time of approval regarding the storage and confidentially of patient data. This statement is subject to change: we have applied, in retrospect, to the ethics committee for approval to share the anonymized data collected. For updates on this request or for requests to access the data, please contact RH (robert.heirene@sydney.edu.au).

## Ethics Statement

The studies involving human participants were reviewed and approved by the Greater Glasgow and Clyde Health Board. The patients/participants provided their written informed consent to participate in this study.

## Author Contributions

PB and JE devised the research questions and study design. PB was responsible for data collection. RH analyzed the data, produced the figures, and managed the project’s Open Science Framework page. RH and PB drafted the manuscript. All authors contributed to the interpretation of findings and to manuscript revisions, approved the final manuscript for submission, and agreed to be accountable for all aspects of the work.

## Conflict of Interest

The authors declare that the research was conducted in the absence of any commercial or financial relationships that could be construed as a potential conflict of interest.

## Footnotes

1 https://sydney.edu.au/brain-mind/resources-for-clinicians/dementia-test.html

2 https://osf.io/m69nj/
