# Supplementary material for: Applicability of the ACE-III and RBANS Cognitive Tests for the Detection of Alcohol-Related Brain Damage
Source: Front Psychol. 2019 Nov 28;10:2636. doi: 10.3389/fpsyg.2019.02636 (PMC6892773; doi:10.3389/fpsyg.2019.02636)
Supplement: Supplementary file 1 [file Data_Sheet_1.PDF]

Jamovi analysis code and outputs (Supplemental Document 1)

Preamble

This document contains the analysis code used in jamovi for all analyses reported in Brown, Heirene, Roderique-Davies, John, & Evans (2019): Applicability of the ACE-III and RBANS cognitive tests for the detection of Alcohol-Related Brain Damage. The code can be used directly in Rj editor in jamovi (or in R). We are currently working on gaining ethical approval to share the raw data collected in the study so that the analysis code presented here can be used alongside data to reproduce all analyses (please contact Rob Heirene for updates on this: robert.heirene@sydney.edu.au).

The jamovi outputs from all analyses are also presented (outputs are unedited, aside from font type and size changes). The code and outputs are divided into four sections: [1] Between group (i.e., AL & ARBD) comparisons of clinical and demographic characteristics [2] Between group comparisons on ACE-III tests scores (including supplementary ANCOVA analyses), [3] Between group comparisons on RBANS tests scores (including supplementary ANCOVA analyses), and [4] Exploratory sub-group and correlational analyses. Benzodiazepines

---

[1] Between group (i.e., AL & ARBD) comparisons of clinical & demographic characteristics

Analyses: *t*-tests & Mann-Whitney-*U*

Comparison: Between AL and ARBD groups' clinical and demographic characteristics

Jamovi code for analysis

```
jmv::ttestIS(
    data = data,
    vars = c(
        "Drinking History Duration",
        "Age",
        "Abstinence (weeks)"),
    group = "Group",
    welchs = TRUE,
    mann = TRUE,
    norm = TRUE,
    eqv = TRUE,
    desc = TRUE)
```

Outcomes

Test of Normality (Shapiro-Wilk)

	W	p
Drinking History Duration	0.951	0.0192
Age	0.979	0.4239
Abstinence (weeks)	0.622	< .0001

Note. A low p-value suggests a violation of the assumption of normality

Test of Equality of Variances (Levene's)

	F	df	p
Drinking History Duration	0.3825	1	0.5388
Age	2.6980	1	0.1061
Abstinence (weeks)	0.0161	1	0.8994

Note. A low p-value suggests a violation of the assumption of equal variances

Group Descriptives

	Group	N	Mean	Median	SD	SE
Drinking History Duration	AL	30	17.7	16.0	11.69	2.13
	ARBD	28	19.6	20.0	9.97	1.88
Age	AL	30	46.1	47.0	8.93	1.63
	ARBD	28	56.9	58.5	7.19	1.36
Abstinence (weeks)	AL	30	73.0	19.5	144.47	26.38
	ARBD	28	112.0	70.5	108.09	20.43

Independent Samples T-Test

		statistic	df	p
Drinking History Duration	Student's t	-0.654	56.0	0.5159
	Welch's t	-0.657	55.6	0.5136
	Mann-Whitney U	354		0.3034
Age	Student's t	-5.046	56.0	<.0001
	Welch's t	-5.084	54.8	<.0001
	Mann-Whitney U	140		<.0001
Abstinence (weeks)	Student's t	-1.157	56.0	0.2522
	Welch's t	-1.169	53.5	0.2478
	Mann-Whitney U	207		0.0009

Analyses: Chi-square

Association: Between group membership and gender

Jamovi code for analysis

```
jmv::contTables(
    data = data,
    rows = "Gender",
    cols = "Group")
```

Outcomes

Contingency Tables

	Group		
Gender	AL	ARBD	Total
Male	19	18	38
Female	11	10	20
Total	30	28	58

χ^2 Tests

	Value	df	p
χ^2	0.0569	1	0.9399
N	58		

Association: Between group membership and evidence of polysubstance

Jamovi code for analysis

```
jmv::contTables(
    data = data,
    rows = "Group",
    cols = "Polysubstance",
    ci = FALSE)
```

Outcomes

Contingency Tables

	Polysubstance		
Group	Polysubstance user	Non polysubstance user	Total
AL	10	20	30
ARBD	1	27	28
Total	11	47	58

χ^2 Tests

	Value	df	p
χ^2	8.35	1	0.0039
N	58		

Association: Between group membership and evidence of head injuries

Jamovi code for analysis

```
jmv::contTables(
        data = data,
        rows = "Group",
        cols = "Head Injury",
        ci = FALSE)
```

Outcomes

Contingency Tables

	Head Injury		
**Group**	**Yes**	**No**	**Total**
AL	1	29	30
ARBD	5	23	28
Total	6	52	58

χ^2 Tests

	Value	df	p
χ^2	3.29	1	0.0695
N	58		

Associations: Between group membership and medication use

Jamovi code for analyses

```
jmv::contTables(
   data = data,
   rows = "Antidepressants and/or anxiolytics",
   cols = "Group")
jmv::contTables(
   data = data,
   rows = "Benzodiazepines",
   cols = "Group")
jmv::contTables(
   data = data,
   rows = "Anti-psychotics",
   cols = "Group")
jmv::contTables(
   data = data,
   rows = "Disulfiram, naltrexone, acamprosate",
   cols = "Group")
```

Outcomes: Antidepressants/ anxiolytics

Contingency Tables

	Group		
**Antidepressants and/or anxiolytics**	**AL**	**ARBD**	**Total**
Taking	17	15	32
Not taking	13	13	26
Total	30	28	58

χ^2 Tests

	Value	df	p
χ^2	0.0561	1	0.8128
N	58		

Outcomes: Benzodiazepines

Contingency Tables

	Group		
Benzodiazepines	AL	ARBD	Total
Taking	2	1	3
Not taking	28	27	55
Total	30	28	58

χ^2 Tests

	Value	df	p
χ^2	0.283	1	0.5948
N	58		

Outcomes: Anti-psychotic medication

Contingency Tables

	Group		
Anti-psychotics	AL	ARBD	Total
Taking	2	3	5
Not taking	28	25	53
Total	30	28	58

χ^2 Tests

	Value	df	p
χ^2	0.301	1	0.5831
N	58		

Outcomes: Alcohol use medication

Contingency Tables

	Group		
Disulfiram, naltrexone, acamprosate	AL	ARBD	Total
Taking	6	2	8
Not taking	24	26	50
Total	30	28	58

χ^2 Tests

	Value	df	p
χ^2	2.01	1	0.1559
N	58		

Association: Between group membership and occupational status

Jamovi code for analysis

```
jmv::contTables(
    data = data,
    rows = "Occupational status",
    cols = "Group")
```

Outcomes

Contingency Tables

	Group		
**Occupational status**	**AL**	**ARBD**	**Total**
Higher	4	4	8
Intermediate	6	7	13
Lower/Unemployed	20	17	37
Total	30	28	58

χ^2 Tests

	Value	df	p
χ^2	0.251	2	0.8818
N	58		

[2] Between group comparisons on ACE-III tests scores

Analyses: *t*-tests & Mann-Whitney-*U*

Comparison: Between AL and ARBD groups

Jamovi code for analysis

Primary analyses (one-tailed)

```
jmv::ttestIS(
  data = data,
  vars = c(
    "ACE Total Score",
    "ACE Attention",
    "ACE Memory",
    "ACE Fluency",
    "ACE Language",
    "ACE Visuospatial"),
  group = "Group",
  welchs = TRUE,
  mann = TRUE,
  hypothesis = "oneGreater",
  norm = TRUE,
  eqv = TRUE,
  meanDiff = TRUE,
  effectSize = TRUE,
  desc = TRUE,
  plots = TRUE)
```

For two-tailed Welch's *t*-test outcomes

```
jmv::ttestIS(
  data = data,
  vars = c(
    "ACE Total Score",
    "ACE Attention",
    "ACE Memory",
    "ACE Fluency",
    "ACE Language",
    "ACE Visuospatial"),
  group = "Group",
  students = FALSE,
  welchs = TRUE,
  meanDiff = TRUE,
  effectSize = TRUE,
  ci = TRUE)
```

Outcomes

Test of Normality (Shapiro-Wilk)

	W	p
ACE Total Score	0.921	0.0010
ACE Attention	0.879	<.0001
ACE Memory	0.978	0.3812
ACE Fluency	0.927	0.0017
ACE Language	0.682	<.0001
ACE Visuospatial	0.901	0.0002

Note. A low p-value suggests a violation of the assumption of normality

Test of Equality of Variances (Levene's)

	F	df	p
ACE Total Score	0.388	1	0.5357
ACE Attention	22.547	1	< .0001
ACE Memory	0.392	1	0.5337
ACE Fluency	0.112	1	0.7386
ACE Language	0.510	1	0.4781
ACE Visuospatial	1.614	1	0.2091

Note. A low p-value suggests a violation of the assumption of equal variances

Group Descriptives

	Group	N	Mean	Median	SD	SE
ACE Total Score	AL	30	89.4	91.0	8.90	1.624
	ARBD	28	78.54	80.0	10.25	1.936
ACE Attention	AL	30	17.1	18.0	1.17	0.213
	ARBD	28	15.04	16.0	2.97	0.562
ACE Memory	AL	30	21.3	22.0	3.98	0.727
	ARBD	28	16.18	16.5	4.57	0.864
ACE Fluency	AL	30	12.0	13.0	2.26	0.412
	ARBD	28	9.79	10.0	2.56	0.483
ACE Language	AL	30	24.3	25.0	2.85	0.521
	ARBD	28	23.46	24.0	1.60	0.302
ACE Visuospatial	AL	30	14.7	15.0	1.29	0.236
	ARBD	28	14.07	14.0	1.84	0.349

Independent Samples T-Tests

		statistic	df	p	Mean difference	SE difference	Cohen's d
ACE Total Score	Student's t	4.32	56.0	<.0001	10.864	2.515	1.135
	Welch's t	4.30	53.6	<.0001	10.864	2.527	1.135
	Mann-Whitney U	149		<.0001	11.00		1.135
ACE Attention	Student's t	3.58 [a]	56.0	0.0004	2.098	0.586	0.941
	Welch's t	3.49	34.6	0.0007	2.098	0.601	0.941
	Mann-Whitney U	225		0.0008	1.00		0.941
ACE Memory	Student's t	4.56	56.0	<.0001	5.121	1.124	1.197
	Welch's t	4.54	53.7	<.0001	5.121	1.129	1.197
	Mann-Whitney U	156		<.0001	5.00		1.197
ACE Fluency	Student's t	3.50	56.0	0.0005	2.214	0.633	0.920
	Welch's t	3.48	54.0	0.0005	2.214	0.636	0.920
	Mann-Whitney U	206		0.0004	2.00		0.920
ACE Language	Student's t	1.31	56.0	0.0980	0.802	0.613	0.344
	Welch's t	1.33	46.2	0.0945	0.802	0.602	0.344
	Mann-Whitney U	239		0.0021	1.00		0.344
ACE Visuospatial	Student's t	1.51	56.0	0.0681	0.629	0.416	0.397
	Welch's t	1.49	48.0	0.0709	0.629	0.421	0.397
	Mann-Whitney U	341		0.1042	5.27e-5		0.397

Note. H_a AL > ARBD

[a] Levene's test is significant ($p < .05$), suggesting a violation of the assumption of equal variances

Independent Samples T-Tests (two-tailed Welch's tests—mean difference scores and their confidence intervals from this table are reported in the paper)

							95% Confidence Interval		
		statistic	df	p	Mean difference	SE difference	Lower	Upper	Cohen's d
ACE Total Score	Welch's t	4.30	53.6	< .0001	10.864	2.527	5.796	15.93	1.135
ACE Attention	Welch's t	3.49	34.6	0.0013	2.098	0.601	0.877	3.32	0.941
ACE Memory	Welch's t	4.54	53.7	< .0001	5.121	1.129	2.857	7.39	1.197
ACE Fluency	Welch's t	3.48	54.0	0.0010	2.214	0.636	0.940	3.49	0.920
ACE Language	Welch's t	1.33	46.2	0.1891	0.802	0.602	-0.409	2.01	0.344
ACE Visuospatial	Welch's t	1.49	48.0	0.1417	0.629	0.421	-0.217	1.47	0.397

Plots

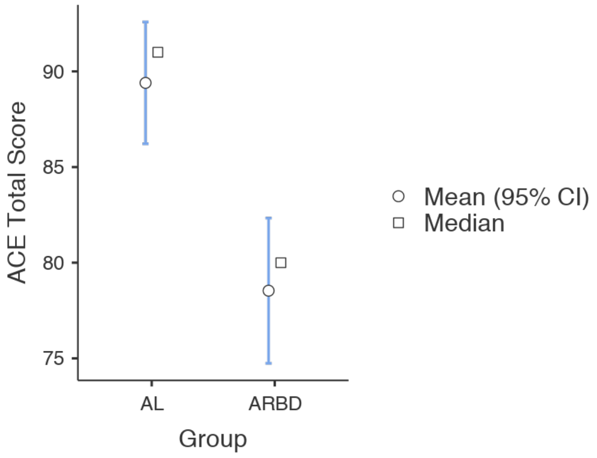

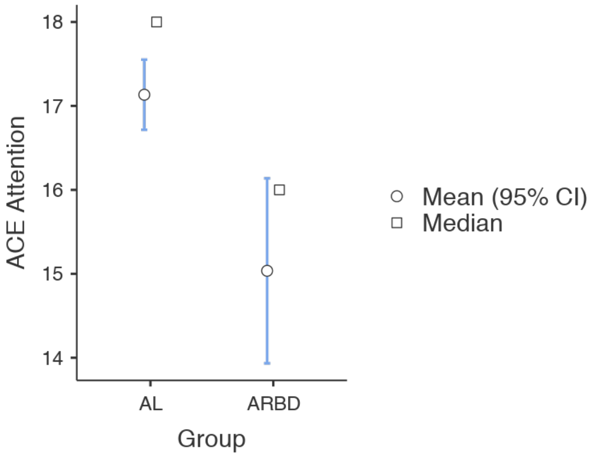

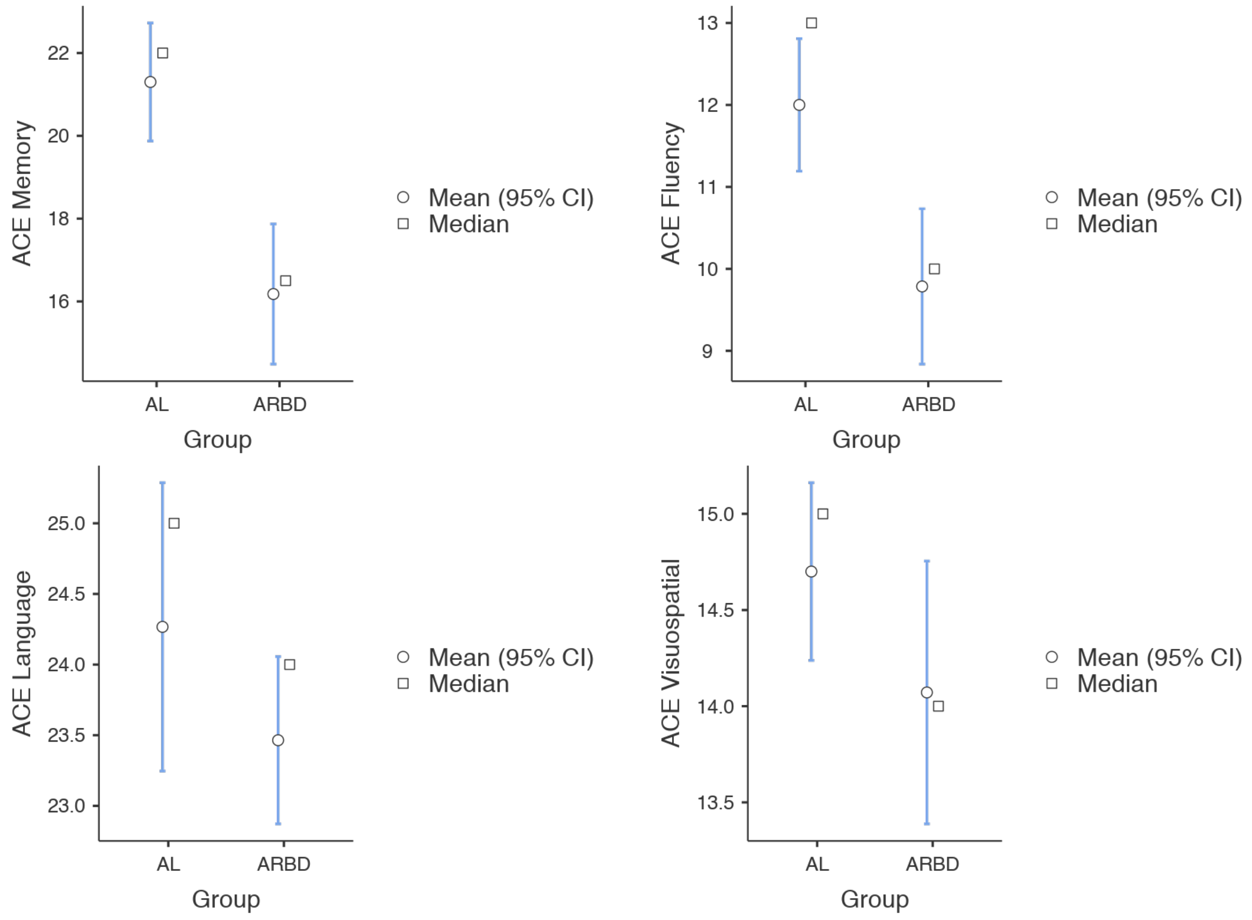

Analyses: ANCOVAs

Comparison: Between group on ACE-III Total score

Jamovi code

```
jmv::ancova(
    data = data,
    dep = "ACE Total Score",
    factors = "Group",
    covs = c(
        "Age",
        "Abstinence (weeks)",
        "Polysubstance"),
    modelTerms = list(
        "Group",
        "Abstinence (weeks)",
        "Polysubstance"),
    effectSize = c("eta", "partEta", "omega"),
    postHocCorr = "none",
    homo = TRUE,
    qq = TRUE,
```

emMeans = list(
  NULL))

Outcomes

Test for Homogeneity of Variances (Levene's)

F	df1	df2	p
0.388	1	56	0.5357

Q-Q Plot

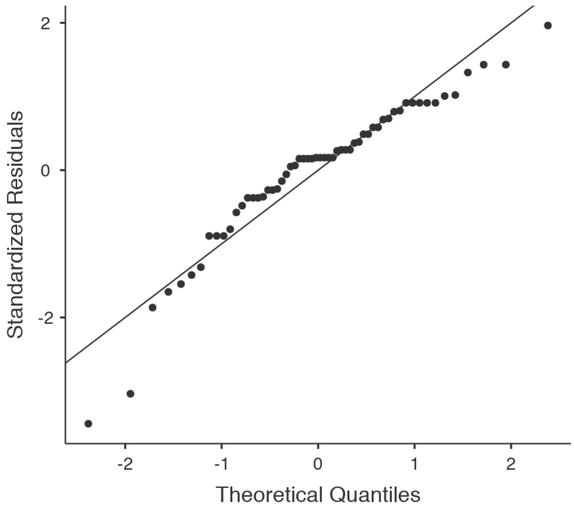

ANCOVA

	Sum of Squares	df	Mean Square	F	p	η^2	η^2p	ω^2
Group	1679.1	1	1679.1	18.588	<.0001	0.249	0.256	0.233
Abstinence (weeks)	26.2	1	26.2	0.290	0.5923	0.004	0.005	-0.009
Polysubstance	159.4	1	159.4	1.764	0.1897	0.024	0.032	0.010
Residuals	4877.9	54	90.3					

Comparison: Between group on ACE-III Attention score

Jamovi code

jmv::ancova(
  data = data,
  dep = "ACE Attention",
  factors = "Group",

```
covs = c(
   "Age",
   "Abstinence (weeks)",
   "Polysubstance"),
modelTerms = list(
   "Age",
   "Group",
   "Abstinence (weeks)",
   "Polysubstance"),
effectSize = c("eta", "partEta", "omega"),
homo = TRUE,
qq = TRUE,
emMeans = list(
   NULL))
```

Outcomes

Test for Homogeneity of Variances (Levene's)

F	df1	df2	p
22.5	1	56	< .0001

Q-Q Plot

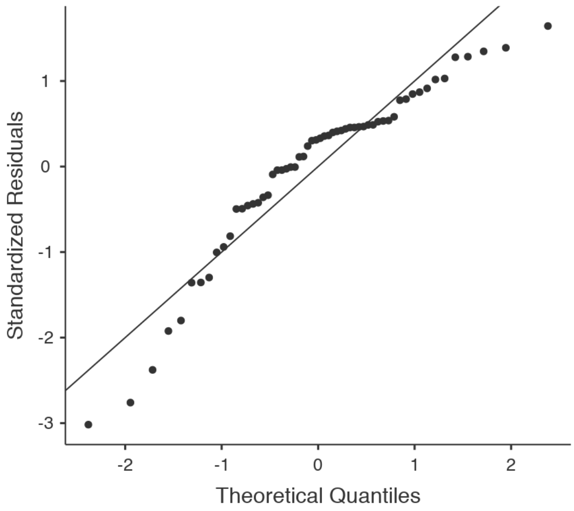

ANCOVA

	Sum of Squares	df	Mean Square	F	p	η^2	η^2p	ω^2
Age	0.242	1	0.242	0.0472	0.8288	0.001	0.001	-0.015
Group	32.862	1	32.862	6.3975	0.0144	0.105	0.108	0.087
Abstinence (weeks)	4.456	1	4.456	0.8675	0.3559	0.014	0.016	-0.002
Polysubstance	3.767	1	3.767	0.7335	0.3956	0.012	0.014	-0.004
Residuals	272.243	53	5.137					

Comparison: Between group on ACE-III Memory score

Jamovi code

```
jmv::ancova(
    data = data,
    dep = "ACE Memory",
    factors = "Group",
    covs = c(
        "Age",
        "Abstinence (weeks)",
        "Polysubstance"),
    effectSize = c("eta", "partEta", "omega"),
    postHocCorr = "none",
    homo = TRUE,
    qq = TRUE,
    emMeans = list(
        NULL))
```

Outcomes

Test for Homogeneity of Variances (Levene's)

F	df1	df2	p
0.392	1	56	0.5337

Q-Q Plot

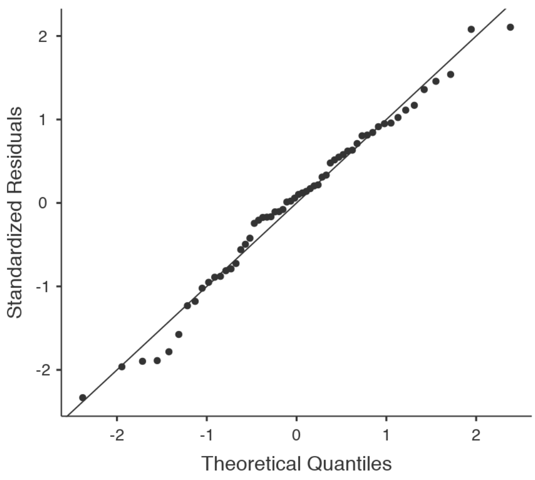

ANCOVA

	Sum of Squares	df	Mean Square	F	p	η^2	$\eta^2 p$	ω^2
Group	265.306	1	265.306	15.4007	0.0003	0.205	0.225	0.189
Age	25.610	1	25.610	1.4867	0.2281	0.020	0.027	0.006
Abstinence (weeks)	0.337	1	0.337	0.0196	0.8893	0.000	0.000	-0.013
Polysubstance	89.314	1	89.314	5.1846	0.0269	0.069	0.089	0.055
Residuals	913.022	53	17.227					

Comparison: Between group on ACE-III Fluency score

Jamovi code

```
jmv::ancova(
    data = data,
    dep = "ACE Fluency",
    factors = "Group",
    covs = c(
        "Age",
        "Abstinence (weeks)",
        "Polysubstance"),
    effectSize = c("omega", "partEta", "eta"),
    postHocCorr = "none",
    homo = TRUE,
    qq = TRUE,
    emMeans = list(
        NULL))
```

Outcomes

Test for Homogeneity of Variances (Levene's)

F	df1	df2	p
0.112	1	56	0.7386

Q-Q Plot

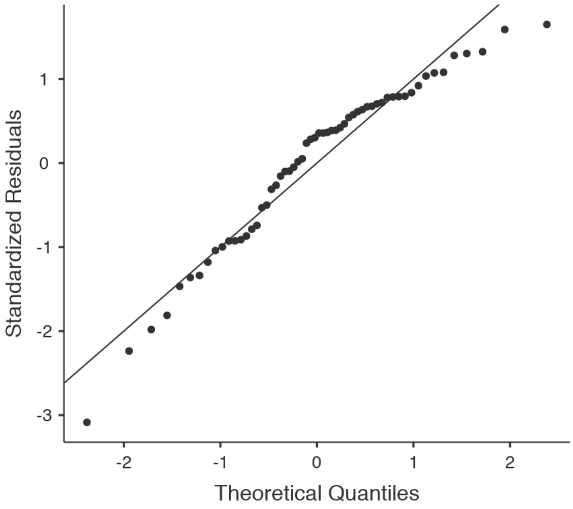

ANCOVA

	Sum of Squares	df	Mean Square	F	p	η²	η²p	ω²
Group	40.1699	1	40.1699	6.84755	0.0115	0.109	0.114	0.092
Age	7.2645	1	7.2645	1.23833	0.2708	0.020	0.023	0.004
Abstinence (weeks)	0.0212	1	0.0212	0.00362	0.9523	0.000	0.000	-0.016
Polysubstance	8.9421	1	8.9421	1.52431	0.2224	0.024	0.028	0.008
Residuals	310.9151	53	5.8663					

Comparison: Between group on ACE-III Language score

Jamovi code

```
jmv::ancova(
    data = data,
    dep = "ACE Language",
    factors = "Group",
    covs = c(
        "Age",
        "Abstinence (weeks)",
        "Polysubstance"),
```

```
effectSize = c("eta", "partEta", "omega"),
postHocCorr = "none",
homo = TRUE,
qq = TRUE,
emMeans = list(
  NULL))
```

Outcomes

Test for Homogeneity of Variances (Levene's)

F	df1	df2	p
0.510	1	56	0.4781

Q-Q Plot

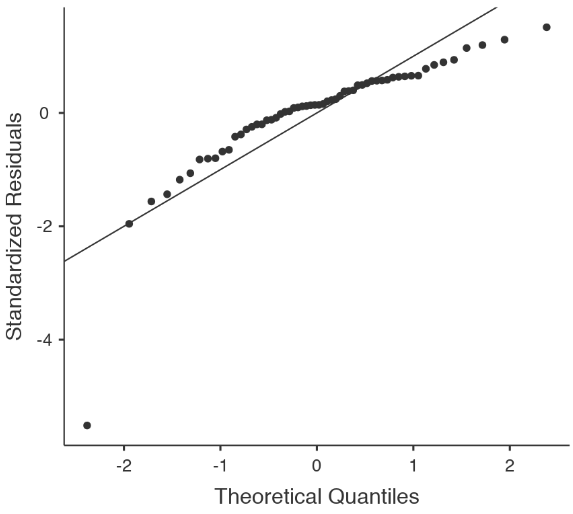

ANCOVA

	Sum of Squares	df	Mean Square	F	p	η^2	η^2p	ω^2
Group	15.31	1	15.31	3.056	0.0862	0.047	0.055	0.031
Age	3.64	1	3.64	0.727	0.3977	0.011	0.014	-0.004
Abstinence (weeks)	2.55	1	2.55	0.509	0.4787	0.008	0.010	-0.007
Polysubstance	38.48	1	38.48	7.679	0.0077	0.118	0.127	0.101
Residuals	265.56	53	5.01					

Comparison: Between group on ACE-III Visuospatial score

Jamovi code

```
jmv::ancova(
    data = data,
    dep = "ACE Visuospatial",
    factors = "Group",
    covs = c(
        "Age",
        "Abstinence (weeks)",
        "Polysubstance"),
    effectSize = c("eta", "partEta", "omega"),
    postHocCorr = "none",
    homo = TRUE,
    qq = TRUE,
    emMeans = list(
        NULL))
```

Outcomes

Test for Homogeneity of Variances (Levene's)

F	df1	df2	p
1.61	1	56	0.2091

Q-Q Plot

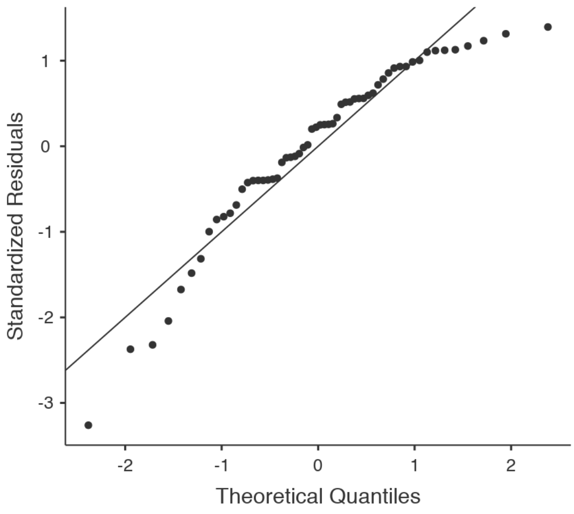

ANCOVA

	Sum of Squares	df	Mean Square	F	p	η^2	η^2p	ω^2
Group	1.0857	1	1.0857	0.4256	0.5170	0.008	0.008	-0.010
Age	0.0261	1	0.0261	0.0102	0.9198	0.000	0.000	-0.017
Abstinence (weeks)	3.5274	1	3.5274	1.3828	0.2449	0.025	0.025	0.007
Polysubstance	2.0546	1	2.0546	0.8054	0.3735	0.014	0.015	-0.003
Residuals	135.1985	53	2.5509					

[3] Between group comparisons on RBANS tests scores

Analyses: *t*-tests & Mann-Whitney-*U*

Comparison: Between AL and ARBD groups

Jamovi code

Primary analyses (one-tailed)

```
jmv::ttestIS(
   data = data,
   vars = c(
      "RBANS Total Score",
      "RBANS Visuospatial/ Constructional",
      "RBANS Attention",
      "RBANS Immediate Memory",
      "RBANS Language",
      "RBANS Delayed Memory"),
   group = "Group",
   welchs = TRUE,
   mann = TRUE,
   hypothesis = “oneGreater”,
   norm = TRUE,
   eqv = TRUE,
   meanDiff = TRUE,
   effectSize = TRUE,
   ci = TRUE,
   desc = TRUE,
   plots = TRUE)
```

For two-tailed Welch’s *t*-test outcomes

```
jmv::ttestIS(
   data = data,
   vars = c(
      "RBANS Total Score",
      "RBANS Immediate Memory",
      "RBANS Visuospatial/ Constructional",
      "RBANS Language",
      "RBANS Attention",
      "RBANS Delayed Memory"),
   group = "Group",
   students = FALSE,
   welchs = TRUE,
   meanDiff = TRUE,
   effectSize = TRUE,
   ci = TRUE)
```

Outcomes

Test of Normality (Shapiro-Wilk)

	W	p
RBANS Total Score	0.989	0.8861
RBANS Immediate Memory	0.981	0.4741
RBANS Visuospatial/ Constructional	0.968	0.1292
RBANS Language	0.933	0.0032
RBANS Attention	0.991	0.9516
RBANS Delayed Memory	0.963	0.0729

Note. A low p-value suggests a violation of the assumption of normality

Test of Equality of Variances (Levene's)

	F	df	p
RBANS Total Score	2.160	1	0.1472
RBANS Immediate Memory	2.550	1	0.1159
RBANS Visuospatial/ Constructional	0.837	1	0.3643
RBANS Language	1.437	1	0.2357
RBANS Attention	0.814	1	0.3707
RBANS Delayed Memory	2.333	1	0.1323

Note. A low p-value suggests a violation of the assumption of equal variances

Group Descriptives

	Group	N	Mean	Median	SD	SE
RBANS Total Score	AL	30	89.8	91.5	16.1	2.93
	ARBD	28	69.2	68.5	11.64	2.20
RBANS Immediate Memory	AL	30	89.1	87.0	18.7	3.42
	ARBD	28	64.9	65.0	13.65	2.58
RBANS Visuospatial/ Constructional	AL	30	96.5	92.0	20.8	3.80
	ARBD	28	83.8	84.0	17.76	3.36
RBANS Language	AL	30	93.4	94.5	11.5	2.09
	ARBD	28	87.0	86.0	7.09	1.34
RBANS Attention	AL	30	91.0	89.5	15.5	2.83
	ARBD	28	80.4	82.0	15.95	3.01
RBANS Delayed Memory	AL	30	91.8	94.0	15.9	2.91
	ARBD	28	62.8	58.0	18.22	3.44

Independent Samples T-Tests

		statistic	df	p	Mean difference	SE difference	95% Confidence Interval Lower	95% Confidence Interval Upper	Cohen's d
RBANS Total Score	Student's t	5.56	56.0	< .0001	20.62	3.71	14.42	Inf	1.462
	Welch's t	5.62	52.8	< .0001	20.62	3.67	14.48	Inf	1.462
	Mann-Whitney U	129		< .0001	21.00		-Inf	-15.00	1.462
RBANS Immediate Memory	Student's t	5.59	56.0	< .0001	24.21	4.33	16.97	Inf	1.469
	Welch's t	5.65	53.0	< .0001	24.21	4.28	17.04	Inf	1.469
	Mann-Whitney U	124		< .0001	25.00		-Inf	-18.00	1.469
RBANS Visuospatial/ Constructional	Student's t	2.51	56.0	0.0076	12.78	5.10	4.25	Inf	0.658
	Welch's t	2.52	55.6	0.0073	12.78	5.07	4.30	Inf	0.658
	Mann-Whitney U	269		0.0095	13.00		-Inf	-4.00	0.658
RBANS Language	Student's t	2.54	56.0	0.0070	6.40	2.52	2.18	Inf	0.666
	Welch's t	2.58	48.8	0.0065	6.40	2.48	2.23	Inf	0.666
	Mann-Whitney U	229		0.0015	7.00		-Inf	-4.00	0.666
RBANS Attention	Student's t	2.55	56.0	0.0068	10.54	4.13	3.63	Inf	0.670
	Welch's t	2.55	55.5	0.0068	10.54	4.14	3.62	Inf	0.670
	Mann-Whitney U	275		0.0119	9.00		-Inf	-3.00	0.670
RBANS Delayed Memory	Student's t	6.47	56.0	< .0001	29.01	4.49	21.51	Inf	1.700
	Welch's t	6.44	53.8	< .0001	29.01	4.51	21.47	Inf	1.700
	Mann-Whitney U	109		< .0001	30.00		-Inf	-20.00	1.700

Note. H_a AL > ARBD

Independent Samples T-Tests (two-tailed Welch's tests—mean difference scores and their confidence intervals from this table are reported in the paper)

		statistic	df	p	Mean difference	SE difference	95% Confidence Interval		Cohen's d
							Lower	Upper	
RBANS Total Score	Welch's t	5.62	52.8	< .0001	20.62	3.67	13.26	28.0	1.462
RBANS Immediate Memory	Welch's t	5.65	53.0	< .0001	24.21	4.28	15.62	32.8	1.469
RBANS Visuospatial/ Constructional	Welch's t	2.52	55.6	0.0147	12.78	5.07	2.62	22.9	0.658
RBANS Language	Welch's t	2.58	48.8	0.0131	6.40	2.48	1.41	11.4	0.666
RBANS Attention	Welch's t	2.55	55.5	0.0136	10.54	4.14	2.25	18.8	0.670
RBANS Delayed Memory	Welch's t	6.44	53.8	< .0001	29.01	4.51	19.98	38.0	1.700

Plots

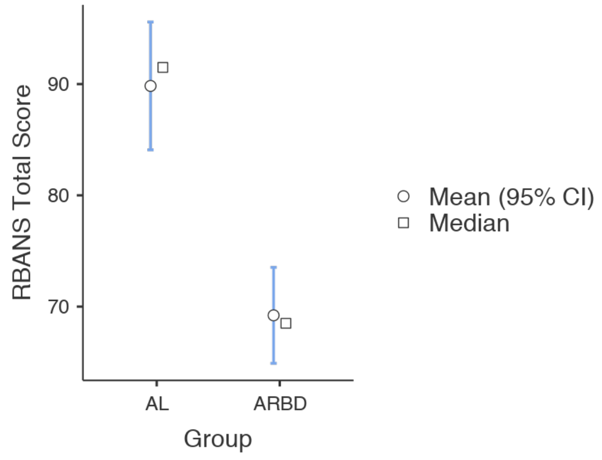

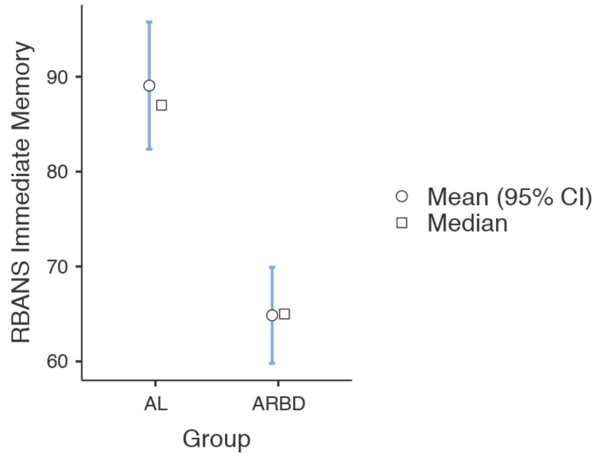

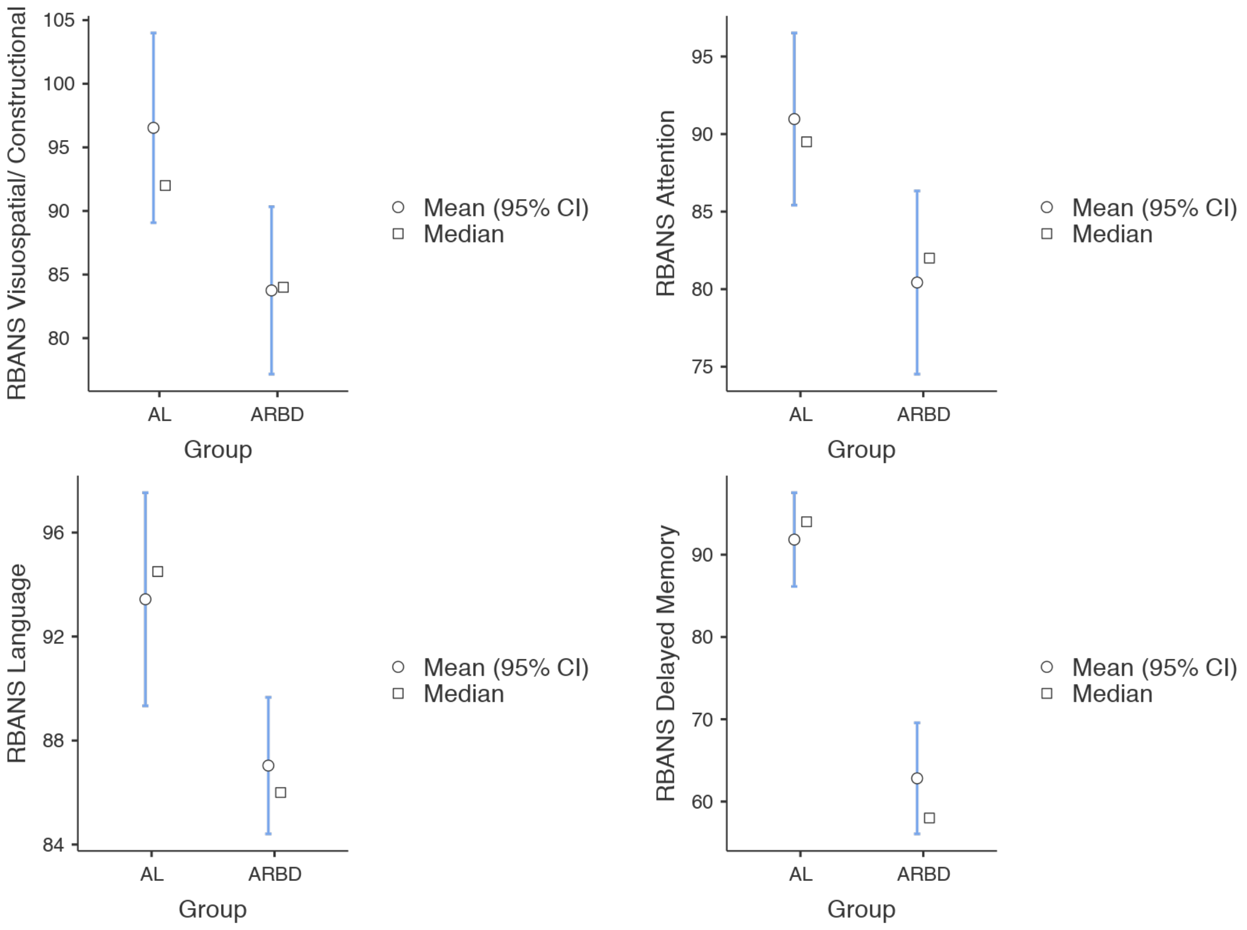

Analyses: ANCOVAs

Comparison: Between group on RBANS total score

Jamovi code

```
jmv::ancova(
    data = data,
    dep = "RBANS Total Score",
    factors = "Group",
    covs = c(
        "Age",
        "Abstinence (weeks)",
        "Polysubstance"),
    effectSize = c("eta", "omega", "partEta"),
    postHocCorr = "none",
    homo = TRUE,
    qq = TRUE,
    emMeans = list(
        NULL))
```

Outcomes

Test for Homogeneity of Variances (Levene's)

F	df1	df2	p
2.16	1	56	0.1472

Q-Q Plot

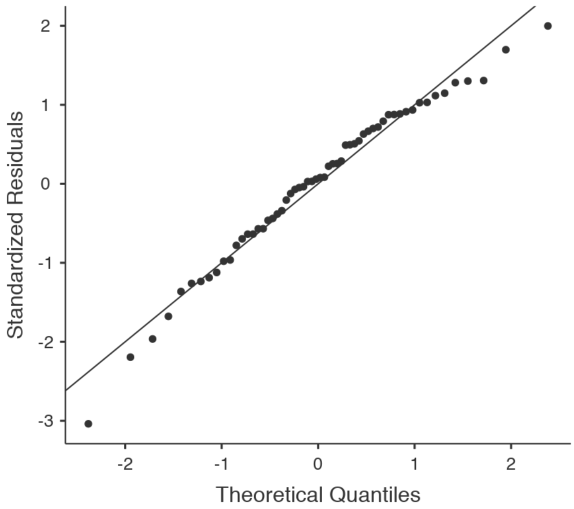

ANCOVA

	Sum of Squares	df	Mean Square	F	p	η²	η²p	ω²
Group	4842	1	4842	30.66	<.0001	0.289	0.366	0.277
Age	675	1	675	4.27	0.0437	0.040	0.075	0.031
Abstinence (weeks)	173	1	173	1.09	0.3006	0.010	0.020	0.001
Polysubstance	2688	1	2688	17.02	0.0001	0.161	0.243	0.150
Residuals	8371	53	158					

Comparison: Between group on RBANS Immediate Memory score

Jamovi code

```
jmv::ancova(
    data = data,
    dep = "RBANS Immediate Memory",
    factors = "Diagnosis",
    covs = c(
        "Age",
```

```
    "Abstinence (weeks)",
    "Polysubstance"),
  effectSize = c("eta", "partEta", "omega"),
  postHocCorr = "none",
  homo = TRUE,
  qq = TRUE,
  emMeans = list(
    NULL))
```

Outcomes

Test for Homogeneity of Variances (Levene's)

F	df1	df2	p
1.20	2	55	0.3081

Q-Q Plot

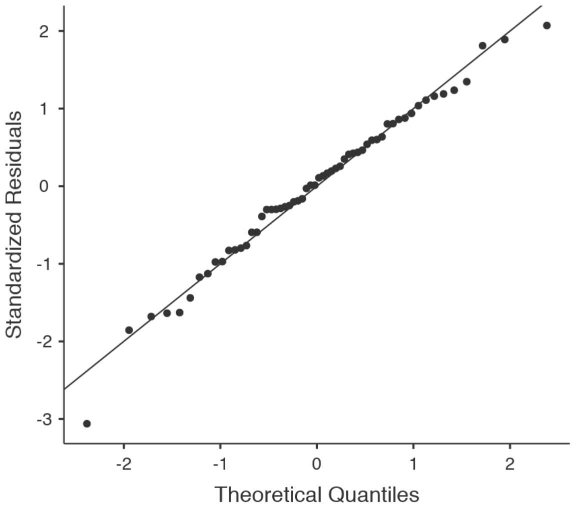

ANCOVA

	Sum of Squares	df	Mean Square	F	p	η^2	η^2p	ω^2
Diagnosis	6722	2	3361	14.83	< .0001	0.290	0.363	0.268
Age	1043	1	1043	4.60	0.0366	0.045	0.081	0.035
Abstinence (weeks)	778	1	778	3.43	0.0696	0.034	0.062	0.024
Polysubstance	2849	1	2849	12.57	0.0008	0.123	0.195	0.112
Residuals	11783	52	227					

Comparison: Between group on RBANS Visuospatial score

Jamovi code

```
jmv::ancova(
    data = data,
    dep = "RBANS Visuospatial/ Constructional",
    factors = "Group",
    covs = c(
        "Age",
        "Abstinence (weeks)",
        "Polysubstance"),
    effectSize = c("eta", "partEta", "omega"),
    postHocCorr = "none",
    homo = TRUE,
    qq = TRUE,
    emMeans = list(
        NULL))
```

Outcomes

Test for Homogeneity of Variances (Levene's)

F	df1	df2	p
0.837	1	56	0.3643

Q-Q Plot

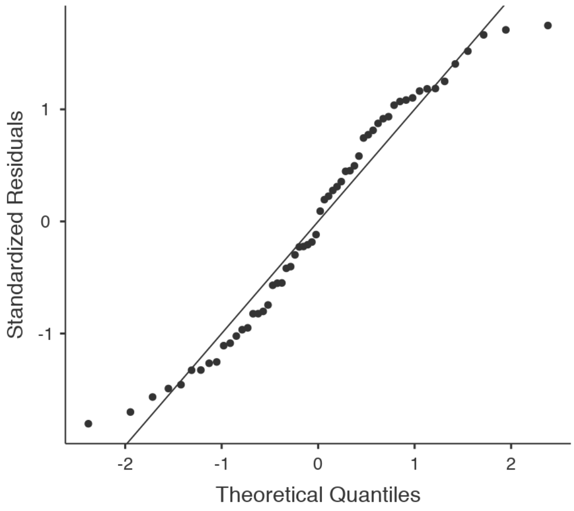

ANCOVA

	Sum of Squares	df	Mean Square	F	p	η^2	η^2p	ω^2
Group	1636.4	1	1636.4	5.4432	0.0235	0.069	0.093	0.056
Age	1717.3	1	1717.3	5.7122	0.0204	0.073	0.097	0.059
Abstinence (weeks)	16.7	1	16.7	0.0557	0.8144	0.001	0.001	-0.012
Polysubstance	4370.4	1	4370.4	14.5375	0.0004	0.185	0.215	0.170
Residuals	15933.3	53	300.6					

Comparison: Between group on RBANS Language score

Jamovi code

```
jmv::ancova(
    data = data,
    dep = "RBANS Language",
    factors = "Group",
    covs = c(
        "Age",
        "Abstinence (weeks)",
        "Polysubstance"),
    effectSize = c("eta", "partEta", "omega"),
    postHocCorr = "none",
    homo = TRUE,
    qq = TRUE,
    emMeans = list(
        NULL))
```

Outcomes

Test for Homogeneity of Variances (Levene's)

F	df1	df2	p
1.44	1	56	0.2357

Q-Q Plot

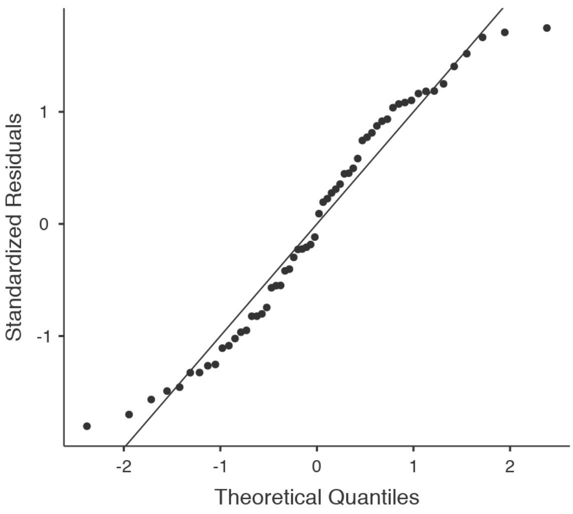

ANCOVA

	Sum of Squares	df	Mean Square	F	p	η^2	η^2p	ω^2
Group	873.748	1	873.748	10.16724	0.0024	0.142	0.161	0.127
Age	0.270	1	0.270	0.00315	0.9555	0.000	0.000	-0.014
Abstinence (weeks)	279.620	1	279.620	3.25376	0.0769	0.046	0.058	0.031
Polysubstance	431.224	1	431.224	5.01787	0.0293	0.070	0.086	0.055
Residuals	4554.693	53	85.938					

Comparison: Between group on RBANS Attention score

Jamovi code

```
jmv::ancova(
   data = data,
   dep = "RBANS Attention",
   factors = "Group",
   covs = c(
      "Age",
      "Abstinence (weeks)",
      "Polysubstance"),
   effectSize = c("eta", "partEta", "omega"),
   postHocCorr = "none",
   homo = TRUE,
   qq = TRUE,
   emMeans = list(
      NULL))
```

Outcomes

Test for Homogeneity of Variances (Levene's)

F	df1	df2	p
0.814	1	56	0.3707

Q-Q Plot

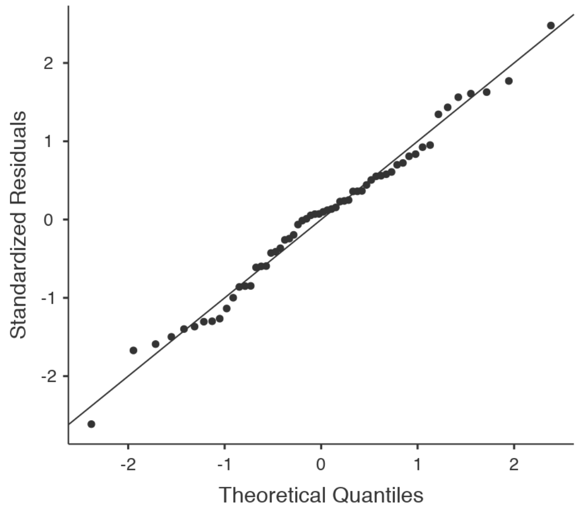

ANCOVA

	Sum of Squares	df	Mean Square	F	p	η²	η²p	ω²
Group	1548.8	1	1548.8	6.4185	0.0143	0.103	0.108	0.085
Age	10.5	1	10.5	0.0436	0.8353	0.001	0.001	-0.015
Abstinence (weeks)	60.7	1	60.7	0.2516	0.6180	0.004	0.005	-0.012
Polysubstance	672.4	1	672.4	2.7865	0.1010	0.045	0.050	0.028
Residuals	12789.4	53	241.3					

Comparison: Between group on RBANS Delayed Memory score

Jamovi code

```
jmv::ancova(
    data = data,
    dep = "RBANS Delayed Memory",
    factors = "Group",
    covs = c(
        "Age",
        "Abstinence (weeks)",
```

```
    "Polysubstance"),
  effectSize = c("eta", "partEta", "omega"),
  postHocCorr = "none",
  homo = TRUE,
  qq = TRUE,
  emMeans = list(
    NULL))
```

Outcomes

Test for Homogeneity of Variances (Levene's)

F	df1	df2	p
2.33	1	56	0.1323

Q-Q Plot

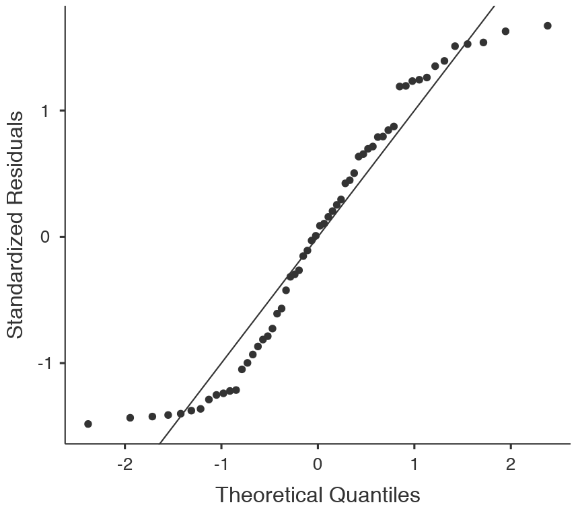

ANCOVA

	Sum of Squares	df	Mean Square	F	p	η²	η²p	ω²
Group	8945	1	8945	32.098	< .0001	0.348	0.377	0.334
Age	351	1	351	1.258	0.2671	0.014	0.023	0.003
Abstinence (weeks)	124	1	124	0.446	0.5069	0.005	0.008	-0.006
Polysubstance	1517	1	1517	5.443	0.0235	0.059	0.093	0.048
Residuals	14770	53	279					

[4] Exploratory sub-group and correlational analyses

Analyses: *t*-tests & Mann-Whitney-*U*

Comparison: Between AL polysubstance users and non-polysubstance users

Jamovi code

```
jmv::ttestIS(
    data = data,
    vars = c(
        "Drinking History Duration",
        "Abstinence (weeks)",
        "Age",
        "RBANS Total Score",
        "ACE Total Score"),
    group = "Polysubstance",
    welchs = TRUE,
    mann = TRUE,
    meanDiff = TRUE,
    effectSize = TRUE,
    ci = TRUE,
    desc = TRUE)
```

Outcomes

Test of Normality (Shapiro-Wilk)

	W	p
Drinking History Duration	0.897	0.0071
Abstinence (weeks)	0.670	< .0001
Age	0.935	0.0650
RBANS Total Score	0.968	0.4851
ACE Total Score	0.832	0.0003

Note. A low p-value suggests a violation of the assumption of normality

Test of Equality of Variances (Levene's)

	F	df	p
Drinking History Duration	1.0556	1	0.3130
Abstinence (weeks)	18.8039	1	0.0002
Age	1.6603	1	0.2081
RBANS Total Score	0.0883	1	0.7686
ACE Total Score	0.8839	1	0.3552

Note. A low p-value suggests a violation of the assumption of equal variances

Group Descriptives

	Group	N	Mean	Median	SD	SE
Drinking History Duration	Polysubstance user	10	18.4	18.0	8.63	2.73
	Non polysubstance user	20	17.4	14.5	13.14	2.94
Abstinence (weeks)	Polysubstance user	10	163.1	61.0	229.46	72.56
	Non polysubstance user	20	28.0	19.5	22.61	5.06
Age	Polysubstance user	10	40.6	40.5	6.06	1.92
	Non polysubstance user	20	48.8	53.0	8.99	2.01
RBANS Total Score	Polysubstance user	10	78.2	81.5	12.87	4.07
	Non polysubstance user	20	95.7	96.0	14.44	3.23
ACE Total Score	Polysubstance user	10	85.5	89.0	11.05	3.49
	Non polysubstance user	20	91.3	92.0	7.14	1.60

Independent Samples T-Test

							95% Confidence Interval		
		**statistic**	**df**	**p**	**Mean difference**	**SE difference**	**Lower**	**Upper**	**Cohen's d**
Drinking History Duration	Student's t	0.228	28.0	0.8212	1.05	4.60	-8.38	10.476	0.0884
	Welch's t	0.262	25.64	0.7956	1.05	4.01	-7.20	9.30	0.0884
	Mann-Whitney U	83.0		0.4660	3.43		-10.0	5.000	0.0884
Abstinence (weeks)	Student's t	2.655 [a]	28.0	0.0129	135.13	50.90	30.87	239.384	1.0282
	Welch's t	1.858	9.09	0.0958	135.13	72.74	-29.18	299.43	1.0282
	Mann-Whitney U	75.5		0.2894	14.22		-169.0	8.000	1.0282
Age	Student's t	-2.594	28.0	0.0149	-8.20	3.16	-14.68	-1.724	-1.0046
	Welch's t	-2.953	25.23	0.0067	-8.20	2.78	-13.92	-2.48	-1.0046
	Mann-Whitney U	41.0		0.0099	-9.00		-15.0	-3.000	-1.0046
RBANS Total Score	Student's t	-3.228	28.0	0.0032	-17.45	5.41	-28.52	-6.377	-1.2503
	Welch's t	-3.358	20.12	0.0031	-17.45	5.20	-28.29	-6.61	-1.2503
	Mann-Whitney U	35.0		0.0045	-17.00		-28.0	-7.000	-1.2503
ACE Total Score	Student's t	-1.758	28.0	0.0897	-5.85	3.33	-12.67	0.966	-0.6809
	Welch's t	-1.523	12.89	0.1519	-5.85	3.84	-14.16	2.46	-0.6809
	Mann-Whitney U	61.0		0.0886	-4.00		-10.0	1.000	-0.6809

[a] Levene's test is significant ($p < .05$), suggesting a violation of the assumption of equal variances

Comparison: Between KS and ARBD

Jamovi code

Filter applied: “group == ‘ARBD’”

```
jmv::ttestIS(
    data = data,
    vars = c(
        "RBANS Total Score",
        "ACE Total Score",
        "Age",
        "Drinking History Duration",
        "Abstinence (weeks)"),
    group = "Diagnosis",
    welchs = TRUE,
    mann = TRUE,
    norm = TRUE,
    eqv = TRUE,
    meanDiff = TRUE,
    effectSize = TRUE,
    ci = TRUE,
    desc = TRUE)
```

Outcomes

Test of Normality (Shapiro-Wilk)

	W	p
RBANS Total Score	0.969	0.5519
ACE Total Score	0.955	0.2680
Age	0.935	0.0840
Drinking History Duration	0.975	0.7156
Abstinence (weeks)	0.820	0.0002

Note. A low p-value suggests a violation of the assumption of normality

Test of Equality of Variances (Levene's)

	F	df	p
RBANS Total Score	3.564	1	0.0703
ACE Total Score	0.230	1	0.6355
Age	1.800	1	0.1914
Drinking History Duration	1.187	1	0.2859
Abstinence (weeks)	2.641	1	0.1162

Note. A low p-value suggests a violation of the assumption of equal variances

Group Descriptives

	Group	N	Mean	Median	SD	SE
RBANS Total Score	ARBD	17	71.5	73.0	12.91	3.13
	KS	11	65.7	66.0	8.79	2.65
ACE Total Score	ARBD	17	79.5	80.0	9.52	2.31
	KS	11	77.1	79.0	11.61	3.50
Age	ARBD	17	56.5	59.0	5.95	1.44
	KS	11	57.5	58.0	9.07	2.73
Drinking History Duration	ARBD	17	21.7	20.0	9.11	2.21
	KS	11	16.2	17.5	10.74	3.24
Abstinence (weeks)	ARBD	17	95.9	70.0	79.43	19.26
	KS	11	136.9	71.0	142.58	42.99

Independent Samples T-Test

							95% Confidence Interval		
		**statistic**	**df**	**p**	**Mean difference**	**SE difference**	**Lower**	**Upper**	**Cohen's d**
RBANS Total Score	Student's t	1.291	26.0	0.2082	5.743	4.45	-3.40	14.89	0.499
	Welch's t	1.400	25.9	0.1733	5.743	4.10	-2.69	14.18	0.499
	Mann-Whitney U	68.5		0.2485	6.000		-15.00	5.00	0.499
ACE Total Score	Student's t	0.593	26.0	0.5584	2.380	4.01	-5.87	10.63	0.229
	Welch's t	0.568	18.4	0.5771	2.380	4.19	-6.41	11.17	0.229
	Mann-Whitney U	86.0		0.7414	1.000		-10.00	6.00	0.229
Age	Student's t	-0.348	26.0	0.7307	-0.984	2.83	-6.80	4.83	-0.135
	Welch's t	-0.318	15.6	0.7545	-0.984	3.09	-7.55	5.59	-0.135
	Mann-Whitney U	88.5		0.8317	-1.000		-6.00	5.00	-0.135
Drinking History Duration	Student's t	1.457	26.0	0.1571	5.508	3.78	-2.26	13.28	0.564
	Welch's t	1.405	18.9	0.1762	5.508	3.92	-2.70	13.71	0.564
	Mann-Whitney U	67.0		0.2121	5.000		-15.00	2.50	0.564
Abstinence (weeks)	Student's t	-0.978	26.0	0.3369	-40.952	41.86	-126.99	45.09	-0.379
	Welch's t	-0.869	14.1	0.3993	-40.952	47.11	-141.95	60.04	-0.379
	Mann-Whitney U	81.5		0.5885	-14.000		-84.00	39.00	-0.379

Analyses: correlations

Correlation matrix: AL group's test scores and characteristics

Jamovi code

Filter applied: "group == 'AL'"

```
jmv::corrMatrix(
    data = data,
    vars = c(
        "Drinking History Duration",
        "ACE Total Score",
        "Age",
        "Abstinence (weeks)",
        "RBANS Total Score"),
    flag = TRUE)
```

Outcomes

Correlation Matrix

		Drinking History Duration	ACE Total Score	Age	Abstinence (weeks)	RBANS Total Score
Drinking History Duration	Pearson's r	—	-0.164	0.071	0.145	-0.124
	p-value	—	0.3875	0.7084	0.4441	0.5123
ACE Total Score	Pearson's r		—	-0.090	-0.045	0.784 ***
	p-value		—	0.6356	0.8133	< .0001
Age	Pearson's r			—	0.027	-0.003
	p-value			—	0.8878	0.9873
Abstinence (weeks)	Pearson's r				—	-0.113
	p-value				—	0.5537
RBANS Total Score	Pearson's r					—
	p-value					—

Note. * p < .05, ** p < .01, *** p < .001

Correlation matrix: ARBD group's test scores and characteristics

Jamovi code

Filter applied: "group == 'ARBD'"

```
jmv::corrMatrix(
```

```
data = data,
vars = c(
  "Drinking History Duration",
  "ACE Total Score",
  "Age",
  "Abstinence (weeks)",
  "RBANS Total Score"),
flag = TRUE)
```

Outcomes

Correlation Matrix

		Drinking History Duration	ACE Total Score	Age	Abstinence (weeks)	RBANS Total Score
Drinking History Duration	Pearson's r	—	-0.023	0.402 *	0.180	-0.092
	p-value	—	0.9066	0.0341	0.3586	0.6419
ACE Total Score	Pearson's r		—	-0.028	-0.253	0.700 ***
	p-value		—	0.8892	0.1935	< .0001
Age	Pearson's r			—	0.146	-0.173
	p-value			—	0.4579	0.3784
Abstinence (weeks)	Pearson's r				—	0.033
	p-value				—	0.8690
RBANS Total Score	Pearson's r					—
	p-value					—

Note. * $p < .05$, ** $p < .01$, *** $p < .001$
